# Seeking Consensus on the Terminology of Value-Based Transformation Through use of a Delphi Process

**DOI:** 10.1089/pop.2019.0093

**Published:** 2020-06-03

**Authors:** Marilyn M. Schapira, Meredith Williams, Alan Balch, Richard J. Baron, Patricia Barrett, Roy Beveridge, Tracie Collins, Susan C. Day, Rushika Fernandopulle, Anders M. Gilberg, Douglas E. Henley, Amy Nguyen Howell, Christine Laine, Christina Miller, Jaewon Ryu, Donald F. Schwarz, Mark D. Schwartz, Jeffrey Stevens, Elizabeth Teisberg, Ken Yamaguchi, Emily Schapira, Rebecca A. Hubbard

**Affiliations:** ^1^ University of Pennsylvania Perelman School of Medicine, Department of Medicine and the Philadelphia VA Medical Center, Philadelphia, Pennsylvania, USA.; ^2^ Humana, Inc., Louisville, Kentucky, USA.; ^3^ National Patient Advocate Foundation, Washington, District of Columbia, USA.; ^4^ American Board of Internal Medicine, Philadelphia, Pennsylvania, USA.; ^5^ National Committee for Quality Assurance, Washington, District of Columbia, USA.; ^6^ University of Kansas School of Medicine Wichita, Wichita, Kanas, USA.; ^7^ Penn Medicine, Penn Internal Medicine University City, Philadelphia, Pennsylvania, USA.; ^8^ Iora Health, Boston, Massachusetts, USA.; ^9^ Medical Group Management Association, Englewood, Colorado, USA.; ^10^ American Academy of Family Physicians, Leawood, Kansas, USA.; ^11^ America's Physician Groups, Los Angeles, California, USA.; ^12^ Annals of Internal Medicine, Philadelphia, Pennsylvania, USA.; ^13^ Health Promotion Council of Southeast Pennsylvania, Philadelphia, Pennsylvania, USA.; ^14^ Geisinger, Danville, Pennsylvania, USA.; ^15^ Robert Wood Johnson Foundation, Princeton, New Jersey, USA.; ^16^ New York University School of Medicine, Department of Population Health, New York, New York, USA.; ^17^ Summit Medical Group, Knoxville, Tennessee, USA.; ^18^ Dell Medical School, Value Institute for Health and Care, The University of Texas at Austin, Austin, Texas, USA.; ^19^ Centene Corporation, St. Louis, Missouri, USA.; ^20^ Memorial Sloan Kettering Cancer Center, Department of Radiation Oncology, New York, New York, USA.; ^21^ University of Pennsylvania Perelman School of Medicine, Department of Biostatistics, Epidemiology, & Informatics, Philadelphia, Pennsylvania, USA.

**Keywords:** delphi technique, population health, health policy, health care reform

## Abstract

Collaboration among diverse stakeholders involved in the value transformation of health care requires consistent use of terminology. The objective of this study was to reach consensus definitions for the terms *value-based care*, *value-based payment*, and *population health*. A modified Delphi process was conducted from February 2017 to July 2017. An in-person panel meeting was followed by 3 rounds of surveys. Panelists anonymously rated individual components of definitions and full definitions on a 9-point Likert scale. Definitions were modified in an iterative process based on results of each survey round. Participants were a panel of 18 national leaders representing population health, health care delivery, academic medicine, payers, patient advocacy, and health care foundations. Main measures were survey ratings of definition components and definitions. At the conclusion of round 3, consensus was reached on the following definition for value-based payment, with 13 of 18 panelists (72.2%) assigning a high rating (7– 9) and 1 of 18 (5.6%) assigning a low rating (1–3): “Value-based payment aligns reimbursement with achievement of value-based care (health outcomes/cost) in a defined population with providers held accountable for achieving financial goals and health outcomes. Value-based payment encourages optimal care delivery, including coordination across healthcare disciplines and between the health care system and community resources, to improve health outcomes, for both individuals and populations.” The iterative process elucidated specific areas of agreement and disagreement for value-based care and population health but did not reach consensus. Policy makers cannot assume uniform interpretation of other concepts underlying health care reform efforts.

## Introduction

The 21^st^ century vision for health care in the United States centers on *value*, taking into account both health outcomes and the costs involved in maintaining and improving health. In 2017, the United States spent 17.2% of gross domestic product (GDP) on health care, significantly more than the next highest spenders (France at 12.3% and Switzerland at 11.5% of GDP).^1^ Yet the United States lags behind other countries in critical metrics of population health.^2,3^ Multiple Centers for Medicare & Medicaid Services (CMS) programs have been implemented to create a shift from volume-based to value-based reimbursement and encourage pursuit of value-based care, including the Medicare Access & CHIP Reauthorization Act of 2015, the Merit-Based Incentive Payment System, the Hospital Value-Based Purchasing Program, and the Physician Value-Based Modifier.^4–8^ The stated aim of CMS' value-based programs is *better care for individuals, better health for populations, lower costs,*^7^ which echoes the Triple Aim articulated by the Institute for Healthcare Improvement (IHI): improving the patient experience of care, improving the health of populations, and reducing the per capita cost of health care.^9^

This new orientation has been described variously as *value in health care,*^10^ *value-based programs,*^7^ *value-based transformation,*^11^ and *transformational shift from volume to value*.^12^ Terms such as *value-based care* and *value-based payment* (or *purchasing*)^7,13^ have arisen as descriptors of the different roles stakeholders play. Achievement of the value-based transformation of health care requires a common understanding between practicing clinicians, health system leaders, and payers regarding how value is defined and measured for purposes of value-based reimbursement. Additionally, public health experts and policy makers must share a common understanding of these terms with those who deliver and pay for health care. The patient's perspective on value also is needed to create a common understanding among stakeholders.^14^

The Institute of Medicine (IOM) Roundtable on Value & Science-Driven Health Care reported that value may be understood differently by different stakeholders.^15^ Value in health care is often tied to the concept of population health,^7,9,13,16,17^ which raises further uncertainty about terminology. The term *population health* now frequently has a broader meaning than it did in 2003 when Kindig and Stoddard proposed their well-known definition, “The health outcomes of a group of individuals including the distribution of such outcomes within the group.”^18^ Kindig has written that the concept is now being applied to the health of patients receiving care in a particular health care organization and that related terms such as *population health management* and *population medicine* have thus emerged.^19^ A 2014 IHI blog post differentiated *population management* (managing and paying for health care services for a defined population) from *population health* (which addresses broader determinants of health) and proposed the phrase *population medicine* as the best expression of how the resources of a health care system can be used to achieve the Triple Aim.^20^ The IOM's Roundtable on Population Health explicitly acknowledges the lack of a commonly held definition of *population health*.^21^ Without a common understanding of terminology, the risk of miscommunication and misaligned endeavors decreases the likelihood of effective transformation.

The Delphi process is a systematic, structured, consensus-forming method using recognized experts who represent different perspectives relevant to the question posed.^22–24^ This method has been used to develop medical appropriateness criteria, clinical guidelines, and to create taxonomies and definitions in the field of medicine.^25–31^ The objective of this study is to use a modified Delphi process to develop practical, broadly applicable definitions for the terms *value-based care, value-based payment*, *population health,* and *population medicine*.

## Methods

### Study design and population

A Delphi panel of national leaders was formed representing diversity in geographic location, sex, work setting, and professional perspective. An in-person meeting was followed by 3 rounds of structured surveys. A scoping review of definitions in the literature for the relevant terms was provided to the Delphi panel prior to the in-person meeting and informed the content of the first survey round (Supplementary Data). The goal of the in-person meeting was to provide a study overview and generate ideas for definitions and definition components. The discussion was audio-recorded and transcribed verbatim.

### Survey content and measures

Each survey round included candidate definitions and definition components to be rated by the panelists. The Round 1 survey used definitions and components obtained from previously conducted scoping reviews (Table 1, Supplementary Table S1, Supplementary Materials Scoping Review). Definitions and components were modified in subsequent rounds based on survey responses. Panelists were asked (1) to rate how well statements defined each term on a scale from 1 (*Not at all*) to 9 (*Extremely well*), and (2) how essential components (words/phrases) would be in a definition, on a scale of 1 (*Not at all*) to 9 (*Extremely essential*), and to provide persuasive comments to support their ratings. Following Rounds 1 and 2, the panelists received a report with their individual ratings and aggregated ratings and comments from the panel. Panelists were encouraged to modify ratings in response to each summary report throughout the 3 survey rounds, with Round 3 considered to be the final results. The study was approved with exempt status by the University of Pennsylvania Institutional Review Board.

**Table 1.** Delphi Survey Definitions and Ratings for Value-Based Care

How well do statements define value-based care? (1-not at all to 9-extremely well)	1–3 n (%)	4–6 n (%)	7–9 n (%)	Total n	Consensus
**ROUND 1: DEFINITIONS OF VALUE-BASED CARE**					
1. In value-based care, achieving high value for patients must become the overarching goal of health care delivery, with value defined as the health outcomes achieved per dollar spent.	0 (0.0)	5 (27.8)	13 (72.2)	18	Yes
2. Value-based care emphasizes the Triple Aim of managing patient populations to achieve quality outcomes, lower costs, and improve the care experience.	1 (5.6)	7 (38.9)	10 (55.6)	18	Half Support
3. Value-based care is care for which payments are tied to achieving cost and quality objectives for patient populations, implying some level of risk for the health care organization.	10 (55.6)	5 (27.8)	3 (16.7)	18	No
4. Value-based care rewards efficient, patient-centered care by paying clinicians based on how well they care for their patients, including keeping people healthy, delivering high-quality care and controlling costs.	6 (33.3)	10 (55.6)	2 (11.1)	18	No
5. Value-based care is providing the right health care at the right prices, stemming rising health care costs, and improving overall health outcomes for individuals, families, and communities.	3 (17.6)	10 (58.8)	4 (23.5)	17	No
**ROUND 2: DEFINITIONS OF VALUE-BASED CARE**					
1. In value-based care, achieving high value for patients is the overarching goal of health care delivery, with value defined as the health outcomes achieved per dollar spent.	0 (0.0)	6 (33.3)	12 (66.7)	18	Approaching
2. Value-based care seeks to achieve high value for patients with value defined in terms of measurable benefits, including quality of care and health outcomes, per dollar spent.	2 (11.1)	6 (33.3)	10 (55.6)	18	Half support
3. Value-based care seeks to achieve high value for patients through holistic care management with value defined in terms of measurable benefits, including quality of care and health outcomes, per dollar spent.	7 (38.9)	8 (44.4)	3 (16.7)	18	No
4. Value-based care seeks to achieve high value for patients with care encompassing wellness, prevention, and treatment, both through clinical care and by addressing social determinants of health. Value is defined in terms of measurable benefits, including the quality of care and health outcomes, per dollar spent.	4 (22.2)	6 (33.3)	8 (44.4)	18	No
5. Value-based care seeks to achieve high value for patients with value defined in terms of measurable benefits, including health outcomes, quality of care, and patient experience, per dollar spent. High value is delivered within the constraints of available resources.	2 (11.1)	11 (61.1)	5 (27.8)	18	No
6. Value-based care seeks to achieve high value for patients through holistic care management with value defined in terms of measurable benefits, including the quality of care and health outcomes, per dollar spent. Value is delivered within the constraints of limited resources and is measured over a time horizon that exceeds individual episodes of care.	5 (27.8)	11 (61.1)	2 (11.1)	18	No
7. Value-based care seeks to achieve high value for patients through holistic care management with value defined in terms of measurable benefits, including the quality of care and health outcomes per dollar spent. Value is measured over a time horizon that exceeds individual episodes of care. Value-based care seeks to improve health on both individual and population levels and address health disparities within a population.	4 (22.2)	9 (50.0)	5 (27.8)	18	No
8. Value-based care seeks to improve value for individuals and populations with value defined as benefits per costs. Cost can be calculated a variety of ways, including from the patient, payer, health care system, or societal perspective. Value is measured over a time horizon that exceeds individual episodes of care.	6 (33.3)	5 (27.8)	7 (38.9)	18	No
9. Value-based care is a health care delivery model that seeks to improve the patient experience, improve the health of populations, reduce the per capita costs of care, and improve the health care provider's experience in the delivery of care.	4 (22.2)	9 (50.0)	5 (27.8)	18	No
10. Value-based care is a model of care that strives to improve health outcomes for individuals and populations through strategic use of resources to obtain optimal health per dollars spent.	2 (11.1)	8 (44.4)	8 (44.4)	18	No
11. Value-based care is a patient-centric model of care that strives to improve the quality of care delivered and health outcomes for individuals and populations through strategic use of resources to obtain optimal health per dollars spent.	1 (5.6)	8 (44.4)	9 (50.0)	18	Half support
**ROUND 3: DEFINITIONS OF VALUE-BASED CARE**					
1. In value-based care, achieving optimal health of both individuals and populations is the overarching goal, with value defined as measurable health outcomes per cost of care.	2 (11.1)	7 (38.9)	9 (50.0)	18	Half support
2. Value-based care seeks to improve the health of both individuals and populations with value defined as measurable health outcomes relative to cost of care.	1 (5.6)	8 (44.4)	9 (50.0)	18	Half support
3. Value-based care seeks to improve the health of patients and populations with value defined as patient-centered health outcomes achieved per cost of care provided.	3 (16.7)	8 (44.4)	7 (38.9)	18	No
4. Value-based care seeks to improve health for both individuals and populations with value defined as measurable health outcomes per cost of care. Value-based care is provided over an extended time horizon.	2 (11.1)	11 (61.1)	5 (27.8)	18	No
5. Value-based care seeks to improve the health of both individuals and populations with value defined as measurable health outcomes per cost of care. Value-based care is provided over an extended time horizon and within the constraints of available resources.	5 (27.8)	11 (61.1)	2 (11.1)	18	No

### Data management and statistical analysis

Survey data were managed using REDCap electronic data capture tools hosted at the University of Pennsylvania.^32^ Drawing on Rand methodology,^22,23^ the study team defined consensus on a definition or definition component a priori as selection by 70% or more of respondents with a rating of 7 to 9 and selection by less than 15% with a rating of 1 to 3. A rating of 7–9 by a panelist is considered an *endorsement* of the definition or definition component, regardless of the number of 1–3 ratings. Panelists were offered $2000 in compensation for their time at the completion of the study.

### Role of funding source

The study was funded by Humana Inc. The Humana project team also provided feedback to investigators at the University of Pennsylvania on the study's design, conduct, and reporting.

## Results

### Panel participants

**FIG. 1.**
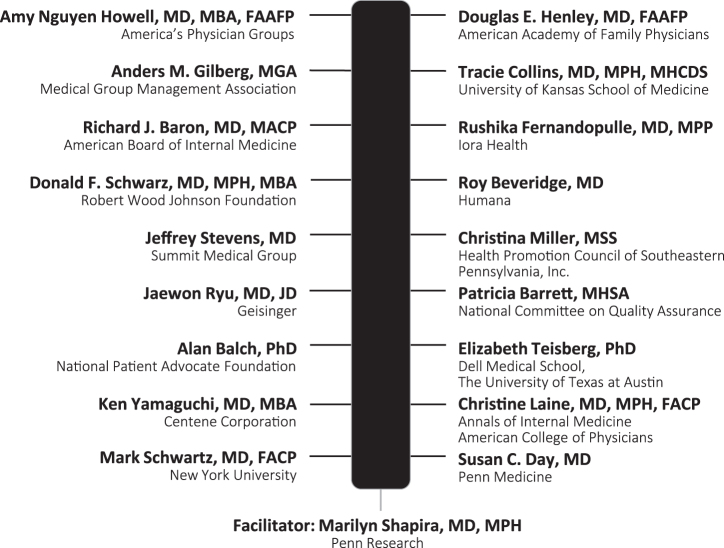
Names and affiliations of Delphi panelists.

Of 21 persons invited, 3 declined, citing conflicts, and 18 either agreed or recommended another leader in their organization. The final panel included 18 leaders who represented diversity of geography, sex, professional perspective, and training (Figure 1). The rate of survey return for each of the 3 survey rounds was 100%.

### Delphi process results for *value-based care*

At the in-person meeting, panelists agreed that value-based care should emphasize value for the patient and incorporate an aspirational goal of improving health. In Round 1, the following definition met a priori criteria for consensus with 13 of 18 (72.2%) panelists providing a rating of 7–9 and 0 panelists providing a rating of 1–3 (Table 1):

In value-based care, achieving high value for patients must become the overarching goal of health care delivery, with value defined as the health outcomes achieved per dollar spent.

In Round 2 this definition no longer met consensus criteria. Panelists commented that the focus of value-based care should expand to individuals (or patients) and populations with ratings for the components *Improve the health of patients* and *Improving the health of individuals and populations* meeting the consensus threshold for being essential to the definition of *value-based care*. The study team further evaluated components pertaining to the numerator and denominator of a value equation. Support was greater for *health outcomes* than for *health benefits* for use in the numerator of the value equation. Including the *Patient experience*, *Preference-aligned patient decisions*, or *What matters most to patients* failed to gain consensus support. The panelists preferred *cost* vs. *dollars spent* as the denominator of the value equation with some commenting on the importance of indirect costs such as lost work time, income loss, adverse outcomes, and emotional stress as important to the value equation (Supplementary Table S1).

The concept that value-based care occurs within the constraints of available resources emerged from the in-person meeting. However, when evaluated as a definition component in Rounds 2 and 3, the phrase had limited support. One panelist noted, “… including this phrase in the definition almost sounds like a cop-out.”

The inclusion of a time horizon over which value-based care is provided and measured was raised at the in-person meeting. However, consensus was not reached on components that conveyed this idea. Panelists commented that the reference to a time frame was too ambiguous and that value can be measured over various time frames.

At the end of Round 3, the 2 definitions with greatest support (endorsed by 50% of panelists) included the components *Achieving (or improving) the health of both individuals and populations* and *Defining value as a measurable health outcome per (or relative to) cost of care* (Table 1).

### Delphi process results for *value-based payment*

At the in-person meeting, panelists indicated that a goal of value-based payment was to support and/or align with the provision of value-based care and reached consensus in Rounds 1 and 2 that a “definition of value” be embedded within the definition of *value-based payment* (Table 2, Supplementary Table S2). Ratings for the definition components of *Accountability* (Round 1) and *Accountability of provider for goals/outcomes/metrics* (Round 2) approached or met a priori criteria for consensus criteria. In contrast, there was limited support for the terms *Reaching quality targets*, *Utilization measures*, *Performance measures*, *Financial incentives for health care providers*, and *Incorporating risk sharing*.

**Table 2.** Delphi Survey Definitions and Ratings for Value-Based Payment

How well do statements define value-based payment? (1-not at all to 9-extremely well)	1–3 n (%)	4–6 n (%)	7–9 n (%)	Total n	Consensus
**ROUND 1: DEFINITIONS OF VALUE-BASED PAYMENT**					
1. Value-based payment rewards value defined as better outcomes and patient experience at a lower cost.	4 (22.2)	10 (55.6)	4 (22.2)	18	No
2. Value-based payments reward providers for the quality and efficiency of care as opposed to the volume of patients treated.	6 (33.3)	8 (44.4)	4 (22.2)	18	No
3. Value-based payment models reward providers who achieve quality and cost targets. Targets can include process measures, health outcomes, and/or utilization measures.	8 (44.4)	8 (44.4)	1 (11.1)	18	No
4. Value-based payment creates a single set of performance measures that spans care settings and applies to a population for which a single group of providers shares accountability.	7 (38.9)	10 (55.6)	1 (5.6)	18	No
5. Value-based payment provides financial incentives to health care organizations based on the patient experience, the premise being that patient experience is a key component of quality of care.	9 (50.0)	9 (50.0)	0 (0.0)	18	No
6. Value-based payment is a shift from volume-based to outcomes-based provider reimbursement. It incorporates risk sharing to incentivize the achievement of high-quality outcomes with the performance of providers measured against specific financial and quality goals.	5 (29.4)	5 (29.4)	7 (41.2)	17	No
**ROUND 2: DEFINITIONS OF VALUE-BASED PAYMENT**					
1. Value-based payment represents a shift from reimbursement for health care based on volume of services to outcomes-based reimbursement. Value-based payment incorporates financial risk-sharing to incentivize the achievement of value (benefits/costs) with the performance of providers measured against specific financial and outcome goals.	2 (11.1)	5 (27.8)	11 (61,1)	18	Approaching
2. Value-based payment represents a shift from reimbursement in for health care based on volume of services to outcomes-based reimbursement. In value-based payment, provider organizations assume a level of risk with respect to financial loss and/or gain and are held accountable for reaching a set of process or health outcome goals in a defined population.	4 (22.2)	7 (38.9)	7 (38.9)	18	No
3. Value-based payment represents a shift from payment for health care based on volume of services to outcomes-based payment in a way that supports value-based care. Value-based payment ties provider payment to the value (benefits/costs) achieved in a defined population over a time frame that exceeds a single episode of care.	3 (16.7)	8 (44.4)	7 (38.9)	18	No
4. Value-based payment rewards value defined as better outcomes achieved more efficiently for more people, leading to alignment of financial success with health care success.	6 (33.3)	6 (33.3)	6 (33.3)	18	No
5. Value-based payment aligns health care reimbursement with the provision of value-based care. Value-based payment represents a shift from reimbursement tied to volume of services provided to reimbursement based on health outcomes achieved at the individual and population level over a period of time longer than a single episode of care.	4 (22.2)	5 (27.8)	9 (50.0)	18	Half support
6. Value-based payment supports value-based care through reimbursement for health care according to predetermined quality, health outcome, and cost goals for a defined population.	7 (38.9)	5 (27.8)	6 (33.3)	18	No
7. Value-based payment supports the delivery of value-based care by providing incentives to provider organizations to allocate and compensate individual providers and teams in a way that enables health care value (benefits/costs) for patients and populations.	6 (33.3)	6 (33.3)	6 (33.3)	18	No
8. Value-based payment supports the delivery of value-based care by providing incentives to allocate resources and compensate individual providers in a way that enables health care value (benefits/costs) for patients and populations. It should be designed to encourage coordination across health care disciplines and between the health care system and community resources.	6 (33.3)	7 (38.9)	5 (27.8)	18	No
9. Value-based payment aligns provider payment with the provision of value-based care in a defined population. The goals of a value-based payment model are to incentivize optimal care delivery, including coordination across health care disciplines and between the health care system and community resources, in order to improve health outcomes for individuals and populations.	4 (22.2)	3 (16.7)	11 (61.1)	18	Approaching
**ROUND 3: DEFINITIONS OF VALUE-BASED PAYMENT**					
1. Value-based payment supports value-based care (health outcomes/costs) in a defined population with providers held accountable for achieving financial goals and health outcomes. Value-based payment represents a shift from reimbursement for health care based on volume of services to outcomes-based reimbursement.	2 (11.1)	7 (38.9)	9 (50.0)	18	Half support
2. Value-based payment aligns reimbursement with achieving value-based care (health outcomes/cost) in a defined population with providers held accountable for achieving financial goals and health outcomes. In value-based payment, outcomes are measured over a specified period of time.	1 (5.6)	8 (44.4)	9 (50.0)	18	Half support
3. Value-based payment aligns reimbursement with achievement of value-based care (health outcomes/cost) in a defined population with providers held accountable for achieving financial goals and health outcomes. Value-based payment encourages optimal care delivery, including coordination across health care disciplines and between the health care system and community resources, to improve health outcomes for both individuals and populations.	1 (5.6)	4 (22.2)	13 (72,2)	18	Yes

In the in-person meeting, panelists voiced that a population health perspective was essential to the definition of *value-based payment*. In the surveys, panelists supported the definition components *Focus on population health* and *Population-level outcomes* with ratings that met or approached consensus criteria, respectively. In contrast, the terms *Lowering costs per capita* and *Increasing capacity to care for more patients* had limited support.

During the in-person meeting, panelists discussed the importance of coordinating across health care settings and partnering with organizations outside the health care system to achieving the goals of value-based payment. In Round 3, ratings for a component with the phrase “coordination across health care disciplines and between the health care system and community resources” approached consensus criteria with 7–9 ratings by 12 out of 18 (66.7%) panelists.

At the end of Round 3, the Delphi panel reached consensus (13 out of 18 [72.2%] rating 7–9 and only 1 [5.6%] rating 1–3) on the following definition of *value-based payment*:

*Value-based payment aligns reimbursement with achievement of value-based care (health outcomes/cost) in a defined population with providers held accountable for achieving financial goals and health outcomes. Value-based payment encourages optimal care delivery, including coordination across health care disciplines and between the health care system and community resources, to improve health outcomes for both individuals and populations*.

Comments in the Round 3 survey included suggestions to refine the consensus definition by shortening the definition and using the term *payment* in place of *reimbursement.*

### Delphi process results for *population health*

The Delphi panel did not reach consensus on a definition of *population health* but results identified key areas of agreement and disagreement (Table 3, Supplementary Table S3). At the in-person meeting, panelists recognized that a population denominator can be determined in many ways and that health includes many measurable domains. However, the panel did not endorse including detail on these elements in the definition.

**Table 3.** Delphi Survey Definitions and Ratings for Population Health

How well do statements define population health? (1-not at all to 9-extremely well)	1–3 n (%)	4–6 n (%)	7–9 n (%)	Total n	Consensus
**ROUND 1: DEFINITIONS OF POPULATION HEALTH**					
1. Population health is the distribution of health outcomes in a population and the health determinants that influence this distribution.	6 (33.3)	5 (27.8)	7 (38.9)	18	No
2. Population health is the health of a group of individuals, which public health agencies define by geography and health delivery systems define by people receiving care (such as all the patients in a particular accountable care organization).	4 (22.2)	11 (61.1)	9 (16.7)	18	No
3. Population health focuses on improving the health of populations with a special emphasis on reducing disparities in health outcomes and improving the value of health care.	1 (5.6)	8 (44.4)	9 (50.0)	18	Half Support
4. Population health is the health of a population (including mortality, quality of life, and functional status) as determined by access to services, quality of care, health behavior, social environment, and the physical environment.	5 (27.8)	8 (44.4)	5 (27.8)	18	No
5. Population health is a conceptual approach to understanding health that has 2 key principles: (1) the need to address factors at multiple levels, integrating social and biologic processes, and (2) an explicit concern with health equity.	4 (22.2)	12 (66.7)	2 (11.1)	18	No
6. Population health is seen in 2 distinct ways: (1) from a public health perspective, populations are defined by the geography of a community (eg, city, county, region, state, or national levels) and (2) from the perspective of the delivery system (individual providers, groups of providers, insurers, and health delivery systems), population health denotes a “panel” of patients served by the organization.	6 (33.3)	8 (44.4)	4 (22.2)	18	No
**ROUND 2: DEFINITIONS OF POPULATION HEALTH**					
1. Population health is the distribution of health outcomes in a population and the health determinants that influence this distribution	1 (5.6)	7 (38.9)	10 (55.6)	18	Half Support
2. Population health is the distribution of measurable health outcomes and the determinants of those outcomes among a group of individuals with the group defined in many ways. Characteristics that define a group might be based on geography, demographic factors, medical conditions, health plan membership, health care provider, or social community. Health is defined as overall well-being across social, mental, and physical health domains.	1 (5.6)	8 (44.4)	9 (50.0)	18	Half Support
3. Population health is the distribution of measurable outcomes among a group of individuals, with the group defined in many ways. Characteristics that define a group can include geography, demographic factors, health conditions, health plan membership, health care provider, or social community. Health is defined as overall well-being across social, mental, and physical domains. Health metrics may include categories such as functional status, quality of life, morbidity, and mortality.	3 (16.7)	6 (33.3)	9 (50.0)	18	Half support
4. Population health is the distribution of measurable health outcomes and the determinants of those outcomes among a group of individuals, with group defined in many ways. Health is systematically measured in the aggregate for the overall population and for subpopulations in order to detect disparities. Optimizing population health in addition to individual health, using a given level of resources, is a goal of value-based care.	3 (16.7)	10 (55.6)	5 (27.8)	18	No
5. Population health is the distribution of measurable health outcomes and determinants of those outcomes among a group of individuals, with group able to be defined in many ways. Health is defined as overall well-being, including social, mental, and physical domains. Determinants of population health include social determinants (education, housing, environmental safety, food), genetic makeup, health behaviors, and access to health care.	3 (16.7)	7 (38.9)	8 (44.4)	18	No
6. Population health is the distribution of measurable health outcomes in a group of individuals with the group defined in many ways.	2 (11.1)	10 (55.6)	6 (33.3)	18	No
7. Population health is the health of a population, including functional status, quality of life, morbidity, and mortality, and the determinants of these outcomes including access to health care services, quality of care, health behavior, genetics, and social and physical environment.	3 (16.7)	8 (44.4)	7 (38.9)	18	No
8. Population health is the distribution of measurable health outcomes among a defined group of individuals and assessment of individual, social, and policy-related determinants of this distribution. The group can be defined in many ways including geography, demographic factors, health care conditions, health plan membership, health care provider, or social community. Health is measured in the aggregate for the overall population and for subpopulations in order to detect disparities. Determinants of population health include social determinants (education, housing, environmental safety, food), genetic makeup, health behaviors, and access to health care.	3 (16.7)	8 (44.4)	7 (38.9)	18	No
**ROUND 3: DEFINITIONS OF POPULATION HEALTH**					
1. Population health is the distribution of measurable health outcomes among a defined group of individuals	1 (5.6)	7 (38.9)	10 (55.6)	18	Half support
2. Population health is the distribution of measurable health outcomes among a defined group of individuals and the determinants of those outcomes	2 (11.1)	10 (55.6)	6 (33.3)	18	No
3. Population health is the distribution of measurable health outcomes among a defined group of individuals and the socioeconomic, environmental, biologic, and behavioral determinants of those outcomes	1 (5.6)	9 (50.0)	8 (44.4)	18	Yes
4. Population health is the distribution of measurable health outcomes among a group of individuals and the determinants of those outcomes. A group is defined by common characteristics such as geography, demographics, health conditions, or health care setting.	4 (22.2)	6 (33.3)	8 (44.4)	18	No
5. Population health is the distribution of measurable outcomes in a defined group of individuals and the social, economic, environmental, and biologic determinants of these outcomes. Health encompasses social, mental, and physical well-being.	2 (11.1)	8 (44.4)	8 (44.4)	18	No

The panel endorsed the component, *A range of determinants such as social determinants (education, housing, environmental safety, food), genetic makeup, health behaviors,* and *access to care* as essential to the definition of *population health*. However, definitions with a reference to determinants of health did not receive consensus ratings. One panelist commented, “Social determinants too often means ‘Not my job.’” Other arguments centered on the appropriate scope of the definition; for example, “The determinants are ‘causes’ while the health outcomes are, to me, the thing itself.”

Ratings for the phrases *Reducing disparities* and *Increasing equity of health* approached consensus criteria in Round 1. However, definitions evaluated (Rounds 1 and 2) that included *disparities* as a measure of population health had limited support. One panelist commented that “To me, the reduction of health disparities is more appropriately included in a definition of population health management rather than a definition of population health.”

In comments from Round 3, a panelist noted the challenge of defining *population health* as a construct distinct from *population health management* stating, that “…‘Population Health’…typically has a modifier ‘Population Health Management’ or context (improving the health of the population) in which it is being asked about so trying to reach consensus on what it means alone is tough.”

A summary of areas of agreement and disagreement that emerged from the Delphi process across all 3 terms is presented in Table 4.

**Table 4.** Summary of Areas of Agreement and Disagreement^*^

General agreement	Disagreement
**Value-based Care**	
Definitions should refer to value for individuals and populations.	Whether time horizon is an essential element and the duration of time over which value should be measured.
“Health outcomes” as opposed to “health benefits” was preferred as an expression of the numerator of the value equation.	Whether to specify that value-based care occurs within the constraints of available resources.
Health outcomes must be “measurable” to allow assessment of value.	Whether to incorporate “patient experience,” “patient-important outcomes,” and related terminology in the definition.
The denominator of the value equation should be “cost” as opposed to “dollars spent.”	Whether the definition should refer to the different ways in which cost can be measured, including indirect costs.
**Value-based Payment**	
The definition should clarify the meaning of value.	Time horizon (similar issues as noted for Value-based care).
Accountability for financial goals and health outcomes is an essential component of the definition.	
Coordination across health care disciplines and between health care system community resources is an essential component of the definition.	
**Population Health**	
The definition should refer to distribution of health outcomes in a population.	Whether the definition should refer to determinants of health, especially social determinants of health.
Population can be defined in a variety of ways (eg, by provider panel, geography, medical diagnosis).	Whether and with what terms the definition should illustrate the different ways of defining populations.
	Whether the definition should specify the domains of health (physical, social, mental) or refer to global measures such as functional status, quality of life, or wellness.

^*^ Reflects both the components that were explicitly rated and ideas that emerged in discussion or as written comments.

## Discussion

The terms *value-based care*, *value-based payment*, and *population health* have gained resonance among stakeholders pursuing the goals articulated by the IHI and implicitly endorsed by CMS: improving the patient experience, controlling health care costs, and improving the health of populations.^7,9^ Health policies and care delivery innovations designed around these concepts are changing the way we evaluate and pay for health care. However, despite their widespread use, the terms lack consensus definitions. The willingness of 18 high-level leaders to participate in this Delphi study is one indication of the widespread recognition of this risk. This study is the first to use a formal consensus process to attempt to create definitions for the related terms of *value-based care*, *value-based payment*, and *population health*.

Through a modified Delphi process the panel came to consensus on a definition for *value-based payment* but not for *value-base care* or *population health*. Collective ratings and comments show where there is a common understanding across stakeholders and where issues of controversy and confusion remain. The panel agreed that a definition of *value-based care* should reflect the aspirational goal of improving health in patients and among populations.

The panelists differed on whether the definition should reflect the patient perspective on outcomes. Although panelists agreed on the goal of value to patients, they recognized that what patients identify as important may differ from the clinician perspective or from evidence-based practice measures, as reflected broadly in discussions on value-base care.^14,29,33^ The panel was divided on using phrases such as *patient experience* and *patient-centered outcomes* in the definition. Response to defining *value-based care*, as well as *population health*, in terms of functional status and quality of life were mixed. Patient-reported outcomes and values are not uniformly captured in electronic health records or included in insurance claims.^8,29^ If the efforts of payers, clinicians, and other health care stakeholders are to be reoriented to broader measures of health, current data systems are not adequate. The denominator of value (cost) also was viewed by most panelists from a broad perspective. Public and private payers cannot assume that the direct costs of health care are all that matter to other stakeholders.

Finally, lack of agreement on the time frame of value measurement hindered consensus on a definition of *value-based care*. Objections stemmed from difficulties in describing this component unambiguously and differences of opinion on the most appropriate measurement interval. If those who provide and pay for health care do not have the same time frame in mind, efforts to improve care at the delivery level will not be consistent with policies.

In contrast to the results for *value-based care*, consensus on the related term of *value-based payment* was achieved, with broad agreement that value-based payment must align with the achievement of value-based care. Support for the phrase *Coordination across health care disciplines and between the health care system and community resources* is a key finding. Some panelists commented that addressing social determinants of health was not the direct responsibility of health care providers but the panel's endorsement of language about partnerships recognizes that modifiable upstream causes of disparities in health must ultimately be addressed for population health to improve.^34^ It may be that consensus on *value-based payment* was easier than on *value-based care* because *value-based-payment* is a concrete, transactional term that describes more familiar experiences. Additionally, the health care system has moved more quickly in transforming payment arrangements than in transforming the way care is provided.^4,6,7,35,36^

The obstacles to consensus for the term *population health* highlight the multiple uses of this term in today's health care policy discussions.^37,38^ A particular challenge was reaching a balance between a concise definition and one that incorporates the complexities of the construct, a challenge that previous Delphi panels and other efforts to clarify definitions also have faced.^25,39,40^ Panelists differed on whether the definition should include determinants and outcomes of health or focus on the measurement of health indicators in a population. Lack of a consensus definition may pose a barrier to value-based reform because population health is a central component of both value-based care and value-based payment initiatives.^10^

The consensus definition used for *value-based payment* refers to the concepts of value-based care and population health in broadly agreed-upon terms. It is encouraging that the panel did agree on several aspects of value-based care and the core concept behind population health (see Table 4), although the panel was unable to agree on specific definitions for these 2 terms. The lack of consensus on definitions for *value-based care* and *population health* as stand-alone terms may reflect the need for definitions to be specific to a given context, considering the community, population, and health care setting in which health care is being delivered and the perspectives of the stakeholders involved. The goal of this Delphi process was to develop functional definitions that could be implemented operationally. The challenge remains to develop functional definitions that all stakeholders can agree on. If leaders can focus on the areas of agreement while keeping differences in mind, a common understanding of the terms may evolve over time. In the interim, it may be of value for organizations to develop internal definitions of *value-based care* and *population health* and to strive for more clarity in external partnerships. A related challenge is to craft definitions that are meaningful and clear to patients but also true to clinicians' and other stakeholders' knowledge about effective care. This will be an important process for policy makers to track; understanding the variety of perspectives on value-based care and population health appears critical.

This study has some limitations. First, the goals were to create concise and broadly applicable definitions. Yet these terms each reflected complex constructs and reaching agreement through such a structured process as the Delphi method felt too restrictive to some panelists. Second, the panel may not have captured all relevant stakeholders. Patients were not on the panel although patient and community advocacy organizations were represented. The panel also lacked participants from government because of potential conflicts of interest between government positions and the Delphi process, which asks for individual judgments based on the sum of an individual's professional experience. Strengths of the process included the diversity among panelists in geography and sex, incorporation of an in-person meeting and 3 rounds of ratings, a 100% participation rate on each survey round, and the use of strict a priori criteria for consensus.

In summary, this study was successful in developing a definition for *value-based payment* that had consensus among a group of experts representing multiple health care sectors. This definition incorporates concepts of value-based care and population health, which validates the goal of seeking definitions for all 3 terms in one study. The panel's inability to reach consensus for the terms *value-based care* and *population health* highlights the challenge of agreeing on practical yet sufficiently rich definitions that can further the cause of health care reform. Policy makers cannot assume that there is a uniform interpretation of the concepts behind value-based health care or population health. Efforts must continue to develop a shared understanding of the meaning of and relationship between these terms so that patients, clinicians, health care systems, payers, and other stakeholders actually achieve health care reform.

## Supplementary Material

Supplemental data

Supplemental data
