# Supplementary material for: Seeking Consensus on the Terminology of Value-Based Transformation Through use of a Delphi Process
Source: Popul Health Manag. 2020 Jun 3;23(3):243–55. doi: 10.1089/pop.2019.0093 (PMC7301322; doi:10.1089/pop.2019.0093)
Supplement: Supplemental data [file Suppl_Data.docx]

Scoping Review of the Terms Value-based care, Value-based payment, Population Health, and Population Medicine

Executive Summary

Two separate literature searches (time frame starting in 2010) were conducted to determine current usage and definitions for the terms to be defined by the Delphi panel. One search focused on *value-based care, value-based payment* and related terms, the other on *population health* and *population medicine.* The websites of key healthcare organizations were also surveyed. Each eligible article was reviewed for definitions or uses of the terms that implied a definition. Those excerpts were also used to identify concepts and words/phrases that could be components of definitions, e.g., patient benefits (health outcomes) as a component of a *value-based care* definition and social determinants of health as a component of a *population health* definition.

The definitions and uses of terms identified in these searches became the pool from which to develop the initial panelist survey for the Delphi process. In the selected articles, the distribution of perspectives (based on principal author affiliation) varied by term; see [Figure A-2](#FigureA2) (p. 21) and [Figure A-3](#FigureA3) (p. 37).

| *Value-based care* and *value-based payment:* Key findings | |
| --- | --- |
| *“Value-based care” -- a buzz word with little clarity?* One of the most common terms in the literature (see [Figure A-1](#FigureA1); (p. 20) but few articles offered insight into a definition.  *Value-based care or high-value care?* References to *value-based care* and *value-based payment* suggested a perspective encompassing the overall care of patients, and often a population perspective, while *high-value care* (“health benefits of an intervention justify its harms and costs” [American College of Physicians]) suggested a focus on individual healthcare decisions.  *The numerator for the value equation is evolving.* Quality of care or process measures predominated as the key measure in combination with cost, but increasingly, patient benefits or health outcomes are being emphasized instead of or in addition to quality.  *Patient experience – making patients happy or enabling them to be responsible?* The language summarized in [Table 4](#Table4) (*p. 19* ) reflects both provider accountability and patient responsibility. Note also the many different ways to think of cost. | ***Example Definitions (see also*** [***Table 1***](#Table1)***; p. 6),*** [***Table 2***](#Table2)***; p. 9, and*** [***Table 3***](#Table3)***; p. 13)***   - *National Committee on Quality Assurance:* The redesigned PCMH process will support the shift to **value-based care**, and aligns reporting requirements with expected MACRA changes . . . a move toward rewarding efficient, patient-centered care by paying clinicians based on how well they care for their patients. That includes keeping people healthy, delivering high-quality care and controlling costs. - *Article on compensation strategies at Mayo Clinic:* . . . **value-based payment** models . . . incorporate quality metrics, provides physicians with regular reports of their performance, and already has resulted in greater physician attention to outcomes, safety, and patient experience. . . . value (defined as better outcomes and patient experience at a lower cost). Current . . . compensation models reward only volume of care, which can conflict with the goal of increasing value. - *The Porter-Teisberg definition of value (see Table 2,* [*Figure 1*](#Figure1)*; p.11):* ***Value in health*** as conceived by Michael Porter and Elizabeth Teisberg was broader and more concrete than most findings in the literature review. |

| *Population health* and *population medicine:* Key findings | |
| --- | --- |
| *The “Triple Aim” concept promoted by the Institute of Healthcare Improvement (IHI) was cited as context for many definitions* – for both *population health* and *population medicine*. See [Table 5](#Table5) (p. 22), [Table 8](#Table8) (p. 32), and [Table 9](#Table9) (p. 34).  *The following familiar definition of ‘population health’ (Kindig & Stoddart. American journal of public health. 2003;93(3):380-383) remains influential and was quoted by many articles:*  The health outcomes of a group of individuals, including the distribution of such outcomes within the group. These groups are often geographic populations such as nations or communities, but can also be other groups such as employees, ethnic groups, disabled persons, prisoners, or any other defined group. The health outcomes of such groups are of relevance to policy makers in both the public and private sectors.  *Population health was often described as a conceptual model* that describes the relationships between determinants of health (including social determinants), interventions designed to improve health (including health policies), and health outcomes (including morbidity, mortality, and well-being).  *‘Population’ was defined by various factors,* often either a geographic area or another commonality within a larger cohort defined by a medical condition or healthcare delivery site. See [Table 5](#Table5) (p. 22), [Table 6](#Table6) (p. 27), [Table 7](#Table7) (p. 31), and [Table 8](#Table8) (p. 32). | ***‘Population medicine’ is much less familiar than ‘population health’.*** The term appeared in only 34 articles, and only five articles provided a useful definition. See [Table 8](#Table8), (p. 32).  ***‘Population medicine’ definitions referred to the impact of healthcare services on population health as in the following definition provided by the IHI (see*** [***Table 5***](#Table5)***, p. 22):*** *Population Medicine* is the design, delivery, coordination, and payment of high-quality healthcare services to manage the Triple Aim for a population using the best resources we have available within the healthcare system.  ***What’s the difference between ‘population* medicine’ *and ‘public health’ or ‘population health’?*** One article (see [Table 8](#Table8), p. 32) identified “focus on system design” as the distinctive feature of population medicine.  ***Population medicine was also discussed in the context of medical education*** *–* teaching physicians to understand medical practice from a population health perspective. |

Literature Scoping Reviews: Select Details

Table of Contents

[*Value-based Care*, *Value-based Payment*, and Related Terms 5](#_Toc506298758)

[Statement of Goals – VBC/VBP Review 5](#_Toc506298759)

[Methods – VBC/VBP Review 5](#_Toc506298760)

[Results – VBC/VBP Review 6](#_Toc506298761)

[Definitions on Websites of Healthcare Organizations 6](#_Toc506298762)

[Literature Search Results 8](#_Toc506298763)

[Definitions of *Value-based Care, Value-based Payment* and Related Terms 9](#_Toc506298764)

[Appendix: *Value-based Care, Value-based Payment* and Related Terms 20](#_Toc506298765)

[Population Health and Population Medicine 22](#_Toc506298766)

[Statement of Goals – Pop Health/Pop Medicine Review 22](#_Toc506298767)

[Methods – Pop Health/Pop Medicine Review 22](#_Toc506298768)

[Results – Pop Health/Pop Medicine Review 22](#_Toc506298769)

[Definitions on Organization Websites 22](#_Toc506298770)

[Literature Search Results 26](#_Toc506298771)

[Definitions of Population Health and Population Medicine 26](#_Toc506298772)

[Appendix: *Population Health* and *Population Medicine* 37](#_Toc506298773)

**List of Tables and Figures**

*Value-based Care, Value-based Payment,* and Related Terms

[Table 1](#Table1). Selected Examples of Terms As Used by National Organizations, (*Value-based Care, Value-based Payment* and Related Terms) (p. 6)

[Table 2](#Table2). Examples of Implied Definitions of *Value-based Care* and *Accountable Care* (p. 9)

[Table 3.](#Table3) Examples of Explicit or Implied Definitions of *Value-based Payment, Value-Based Purchasing, Value-Based Reimbursement* and *Value-Based Contracting* (p. 13)

[Table 4.](#Table4) Specific Language Related to Potential Definition Components (*Value-based Care, Value-based Payment,* and Related Terms) (p. 19)

[Figure 1.](#Figure1) The Outcome Measures Hierarchy (Michael Porter) (p. 11)

[Table A-1.](#TableA1) Journals Included in the Review (p. 20)

[Figure A-1](#FigureA1). Number of Articles with Terms Related to Value-based Care or Value-based Payment in Title or Abstract, 2010-2016, Any Journal (p. 20)

[Figure A-2](#FigureA2). Mix of Author Perspectives in Articles Selected for Definitions/Uses of *Value-based Care, Value-based Payment* and Related Terms (p. 21)

*Population Health* and *Population Medicine*

[Table 5](#Table5). Definitions Identified on Organizational Websites, *Population Health* and *Population Medicine* (p. 22)

[Table 6](#Table6). *Population Health* Definitions and Perspectives (p. 27)

[Table 7](#Table7). *Population Health* Definition Components and Examples (p. 31)

[Table 8](#Table8). *Population Medicine* Definitions and Perspectives (p. 32)

[Table 9](#Table9). *Population Medicine* Definition Components and Examples (p. 34)

[Table 10](#Table10). Definitions of *Population Health Management* (p. 34)

[Figure 2](#Figure2). Framework for Population Health (Kindig) (p. 25)

[Table A-2](#TableA2). Journals for Population Health Search (p. 37)

[Figure A-3](#FigureA3). Perspective of Articles Selected for Definitions/Uses of *Population Health* and *Population Medicine* (p. 37)

# *Value-based Care*, *Value-based Payment*, and Related Terms

## Statement of Goals – VBC/VBP Review

This review was originally conducted with the goal of publication in mind and sought to assess the need for the Delphi panel project. The primary goal was to identify the most common definition(s), stated or implied, for *value-based care, value-based payment* and related terms, in the context of professional services for adult primary or geriatric care. Secondary goals were to a) determine the frequency with which terms of interest appear in the literature and b) assess whether individual terms are used in a consistent manner in the literature. This summary focuses on a sample of the texts offering explicit or implied definitions, summarizes the elements reflected in the entire set of selected texts, and summarizes perspectives (lead author affiliations).

## Methods – VBC/VBP Review

A search of websites for healthcare organizations revealed several terms similar to *value-based care* and *value-based payment*, resulting in nine terms of interest on which to base the literature search: *accountable care*, *high-value care*, *value-based care*, *value-based contracting, value-based insurance design (VBID), value-based payment, value-based purchasing, value-based reimbursement, value-based pricing.*

A systematic search of PubMed was conducted on April 13, 2017 according to these parameters:

- Publication date January 1, 2010 or later.
- The nine terms of interest as search terms, bracketed by quotation marks
- Limited to English language

To make the scope of the project manageable, a set of 24 journals was defined from which to select articles for detailed review. Journal inclusion criteria were designed to identify the journals most likely to be read by primary care physicians or geriatricians, health policy experts, quality improvement experts, and healthcare executives, taking into account journal impact factor and other considerations (see [Table A-1](#FigureA1) in the Appendix, (p. 20). The authors additionally reviewed abstracts of articles from journals that were omitted but were represented by substantial numbers of articles in the initial search results. This methods check did not reveal any evidence of definitions that were different from the range of definitions included in the analysis.

Articles were excluded if they met at least one of the following six exclusion criteria: narrow clinical focus, non-U.S. perspective, perspective of a specialty not typically associated with population health initiatives, book or other nonjournal publication, use of terms outside the context of professional services (application to purchase of drugs or devices), or general irrelevance. The abstract and full-text of each article were examined for any explicit definitions or illustrative uses of the terms of interest. For each text selected, the presence of any of the following elements was noted: cost, contrast with volume, benefits, harms, quality, patient experience, and defined population. These potential components of a definition were identified *a priori* through review of healthcare organization websites. Article selection and text abstraction were performed through dual review and reconciliation, if needed, by another author.

## Results – VBC/VBP Review

### Definitions on Websites of Healthcare Organizations

Table 1 shows the most relevant definitions found on organizational websites. Only one online organizational source each provided an explicit definition of *value-based care* (American Medical Association) or *value-based payment* (American Academy of Family Physicians).

**Table1. Selected Examples of Terms As Used by National Organizations (*Value-based Care, Value-based Payment* and Related Terms)**

| **Organization** | **Definitions** |
| --- | --- |
| **CMS** | |
| ***Better Care, Smarter Spending. Healthier People*** | HHS has adopted a framework that categorizes health care payment according to how providers receive payment to provide care.  •category 1—fee-for-service with no link of payment to quality  •category 2—fee-for-service with a link of payment to quality  •category 3—alternative payment models built on fee-for-service architecture  •category 4—population-based payment  **Value-based purchasing i**ncludes payments made in categories 2 through 4. Moving from category 1 to category 4 involves two shifts: (1) increasing accountability for both quality and total cost of care and (2) a greater focus on population health management as opposed to payment for specific services.  <https://www.cms.gov/Newsroom/MediaReleaseDatabase/Fact-sheets/2015-Fact-sheets-items/2015-01-26-3.html> |
| ***Medicare Advantage Value-Based Insurance Design Model*** | **Value-Based Insurance Design (VBID)** generally refers to health insurers’ efforts to structure enrollee cost sharing and other health plan design elements to encourage enrollees to use high-value clinical services – those that have the greatest potential to positively impact enrollee health.  <https://www.cms.gov/Newsroom/MediaReleaseDatabase/Fact-sheets/2015-Fact-sheets-items/2015-09-01.html> |
| ***What are value-based programs?*** | **Value-based programs** reward health care providers with incentive payments for the quality of care they give to people with Medicare. These programs are part of our larger quality strategy to reform how health care is delivered and paid for. Value-based programs also support our three-part aim [otherwise known as the Triple Aim]:  Better care for individuals - Better health for populations - Lower cost  <https://www.cms.gov/Medicare/Quality-Initiatives-Patient-Assessment-Instruments/Value-Based-Programs/Value-Based-Programs.html> |
| **CMS report to Congress: *Alternative Payment Models & Medicare Advantage*** | MACRA created a new incentive for providers to engage in **value-based contracting** with all payers, . . . bonus payment to providers who have entered certain types of **value-based payment** arrangements.  <https://www.cms.gov/Medicare/Medicare-Advantage/Plan-Payment/Downloads/Report-to-Congress-APMs-and-Medicare-Advantage.pdf> |
| **Agency for Healthcare Research** | |
| **Quality Innovations Exchange: *Value-Based Reimbursement* *Structures*** | **Value-based reimbursement** models aim to improve quality and reduce costs by creating incentives for providers to deliver high-quality rather than volume-driven care.  <https://innovations.ahrq.gov/issues/2013/07/03/value-based-reimbursement-structures> |
| **Physician Groups** | |
| **AMA: *Preparing Your Practice for Value-based Care*** | Unlike traditional fee-for-service care models . . . **value-based care** is intended to at least partially link payments to patients’ health outcomes and/or quality of care.  <https://www.stepsforward.org/Static/images/modules/35/downloadable/Preparing_your_practice_for_value_based_care.pdf> |
| **American Academy of Family Physicians: *Value-based Payment*** | **Value Based Payment (VBP)** is a concept by which purchasers of health care . . . hold the health care delivery system at large . . . accountable for both quality and cost of care. . . VBP's aim is to promote enhanced population health management that should result in the improvement of health and/or systemic cost containment or reduction . . . The most notable difference with **value-based contracts** is exposure of physicians to performance risk, or utilization of unnecessary services.  <https://www.aafp.org/about/policies/all/value-based-payment.html> |
| **American College of Physicians: *High Value Care*** | The **High Value Care Committee** (HVCC) develops recommendations and resources focused on High Value Care that assist physicians to provide the best possible care to their patients while simultaneously reducing unnecessary costs to the healthcare system.  <https://www.acponline.org/clinical-information/high-value-care> |
| **Practice Management Groups** | |
| **American Medical Group Association: August 21, 2017 News Release** | “If a practice participates in **value-based care models**, it is a group endeavor. They succeed or not as one. Differentiating by individual clinician undermines this concept and would make participation in these programs needlessly complex for provider organizations.”  <http://www.amga.org/wcm/AboutAMGA/News/2017/20170821.aspx> |
| **MGMA: *Value-Based Management: Compensation Planning* *for Value-Based Models of Care*** | The ongoing shift from fee-for-service reimbursement to **value-based payment and population health models** will change clinician and staff compensation models over time.  <https://www.mgma.com/healthcare-consulting/hot-topics/consulting-articles/pamela-ballou-compensation-planning-for-value-based-models-of-care> |
| **Other Groups** | |
| **NCQA: *Benefits of NCQA Patient-Centered Medical Home Recognition*** | The redesigned PCMH process will support the shift to **value-based care**, and aligns reporting requirements with expected MACRA changes. . .This [MACRA] represents a move toward rewarding efficient, patient-centered care by paying clinicians based on how well they care for their patients. That includes keeping people healthy, delivering high-quality care and controlling costs.  <http://www.ncqa.org/Portals/0/Programs/Recognition/PCMH/NCQA1005-1016_PCMH%20Evidence_Web.pdf> |
| **Robert Wood Johnson Foundation: *Health Care Cost and Value*** | RWJF is engaging health care providers, policymakers, and consumer groups in efforts to provide the right health care at the right price, stem rising health care costs, and improve overall health outcomes for individuals, families and communities.  <https://www.rwjf.org/en/our-focus-areas/topics/health-care-cost-and-value.html> |

### Literature Search Results

The PubMed search yielded a total of 1,253 articles. Exclusion of articles by journal left 985 articles, and application of the additional exclusion criteria resulted in 59 unique articles as sources of relevant and illustrative text. None of the articles using the terms *high-value care, VBID, or value-based pricing* was considered eligible for developing definitions of *value-based care* or *value-based payment*.

Whereas the texts selected for illustration of the term *value-based care* reflect a global perspective, in terms of scope of care, use of the term *high-value care*, frequently by authors representing the American College of Physicians, reflect a focus on an assessment of value one healthcare service at a time. Two examples from the literature further illustrate this single healthcare service, decision-specific perspective:

“Ultimately, then, high-value care is care for which the evidence suggests maximal benefit and minimal harm in the context of a particular patient’s values and priorities.” (Korenstein D. Patient perception of benefits and harms: the Achilles heel of high-value care. *JAMA Intern Med.* 2015;175(2):287-288.)

“The American College of Physicians’ definition for high-value care stipulates that the health benefits of an intervention justify its harms and costs.” (Qaseem A, Alguire P, Dallas P, et al. Appropriate use of screening and diagnostic tests to foster high-value, cost-conscious care. *Ann. Intern. Med.* 2012;156(2):147-149.)

*Value-based insurance design* was found to apply nearly exclusively to patient copays for drugs, and *value-based* *pricing* was found to apply almost exclusively to patient copays for or payer purchase of drugs and to a lesser extent, devices. None of the articles applied these terms to reimbursement for professional services.

### Definitions of *Value-based Care, Value-based Payment* and Related Terms

The following tables display *representative* texts selected from each article and the perspective of the authors (typically derived from author affiliations as identified in the article).

***Value-based Care, Accountable Care***

Results: Ten (10) articles were found to have text suggesting definitions of *value-based care* or *accountable care* (outside the context of ACOs).

Overview: The number of useful uses of the term *value-based care* was surprisingly small, given the high frequency with which the term appeared in search results (see [Figure A-1](#FigureA1), (p. 20). As shown by *representative* selections in [Table 2](#Table2), usage of the term typically referred to *cost* and *quality*. Only one selection implied that *benefits* (health outcomes) should define value. No selections referred to *harms.* Three selections referred to *patient experience* but did not define the term. A few texts referred to population but did not offer definitions. For additional context, Table 2 also includes definitions of *value in healthcare* offered by business professors Michael Porter and Elizabeth Teisberg.

**Table 2. Examples of Implied Definitions of *Value-based Care* and *Accountable Care***

| **First Author** | **Explicit or implied Definition** | **Perspective** |
| --- | --- | --- |
| **Patel**  **2012** | Health care value, defined as the health outcomes achieved per dollars spent. An essential component of the value definition is that, while it incorporates cost, it is not only about cost. Comparative effectiveness research has arisen as a tool for helping clinicians to assess the value of medical interventions. . . . Value for a patient is to determine if it has been validated through evidence-based medicine from rigorous research trials or if it has been used despite weaker evidence. . . . Identifiable variability between individuals or outcomes in tests and diagnostics can lead to dramatic differences in achievable value from a medical intervention. | Large multispecialty group (Cleveland) |
| **Salmon**  **2012** | Under the accountable care model, provider groups or affiliations assume responsibility for the quality and cost of care for the patient populations they serve, sharing in any savings generated if defined quality targets are met. | Private payer (Cigna) |
| **Bunkers**  **2016** | To prepare for the healthcare industry's transition to value-based care, Mayo Clinic Health System implemented a new, value-focused physician compensation plan as part of a larger initiative aimed at system wide clinical integration. . . . This new transformational model would align with evolving payment models in which value, not volume of care, is rewarded, resulting in accessible, higher-quality care, at lower cost, for a better patient experience. | Large multispecialty group (Mayo) |
| **Chatterjee 2015** | . . . patient experience measures should play a critical role in how we judge high-quality, value-based care. | Public health (academic) |
| **Combes**  **2015** | . . . governing boards are wrestling with is how to successfully transition to value based models of care delivery and payment. Many organizations are not yet ready to have their payments tied to achieving cost and quality objectives for patient populations and assuming some level of risk. | Hospital |
| **Sanghavi 2015** | To ensure value-based health care, payers should be clear about the goal of prevention, which is long-term improvement in meaningful health outcomes. . . . Yet the ideal value-based payment would reward the outcome—in this example, lower rates of tobacco use—rather than various process measures. | CMS |
| **Emanuel 2016** | Application and rigorous evaluation of infrastructure changes and incentives are needed to design payment systems that incentivize high-quality, cost-conscious care. | Health policy/behavioral economics (academic) |
| **Parikh**  **2016** | With the advent of accountable care, the health care organizations that succeed will be those that deliver high value. . . . United States health care costs are twice as high as spending in most industrialized countries. One key opportunity for health systems to improve value is by limiting overuse of costly resources, in part by focusing these resources toward high-risk patient groups. | Department of Medicine (academic) |
| **Modes**  **2016** | There is much work to be done to improve the value of care we deliver, but creating a culture of cost consciousness by including subspecialty champions in practice changes and both residents and faculty in educational initiatives is a first step toward sustained success. | Medical education |
| **Meyer**  **2017** | Value-based care emphasizes the Triple Aim of managing patient populations to achieve quality outcomes, lower costs, and to improve the care experience . . . Institutions educating health care workers must provide appropriate education and training to students to prepare them to be leaders in population health management in the value-based care environment. | Health policy (academic) |
| **Porter and Teisberg 2007*** | A value-based system is grounded in 3 simple principles: (1) the goal is value for patients, (2) care delivery is organized around medical conditions and care cycles, and (3) results are measured. | Business School (academic) |
| **Porter 2010*** | Achieving high value for patients must become the overarching goal of health care delivery, with value defined as the health outcomes achieved per dollar spent. | Business School (academic) |

*The two articles by Porter/Teisberg were not actually identified in the literature search because none of the exact terms of interest are included in the articles, and the 2007 article was outside the search timeframe. They are presented here because these articles and the original book (Porter & Teisberg, 2006) are widely cited. The following graphic (**Figure 1)** comes from Porter (2010).


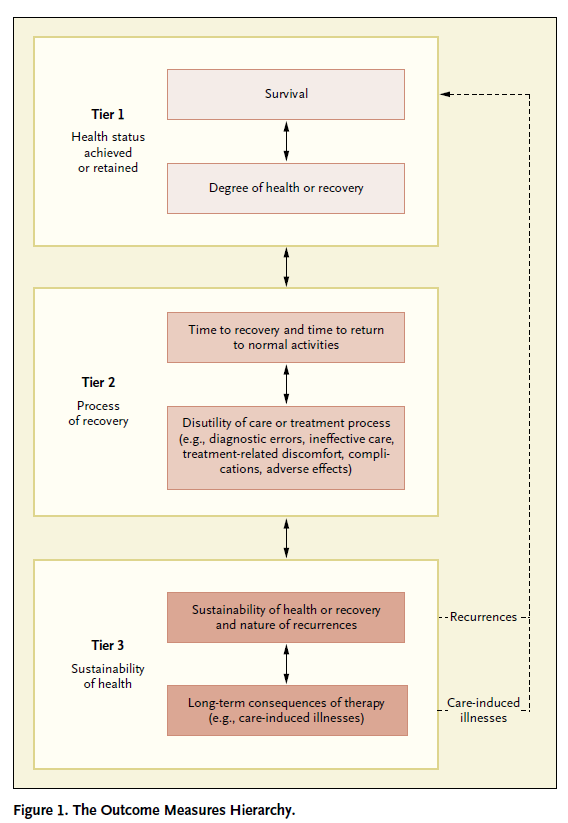


References, *Value-based Care*

Bunkers B, Koch M, Lubinsky J, Weisz JA, Whited B. Value-based physician compensation: a link to performance improvement. *Healthc Financ Manage.* 2016;70(3):52-58.

Chatterjee P, Tsai TC, Jha AK. Delivering value by focusing on patient experience. *Am J Manag Care.* 2015;21(10):735-737.

Combes JR, Totten MK. Leadership for value-based care. Four areas of knowledge and skills must be mastered to make the move from fee for service. *Healthc Exec.* 2015;30(3):74, 76-77.

Emanuel EJ, Ubel PA, Kessler JB, et al. Using Behavioral Economics to Design Physician Incentives That Deliver High-Value Care. *Ann Intern Med.* 2016;164(2):114-119.

Meyer M. Qualifications and Competencies for Population Health Management Positions: A Content Analysis of Job Postings. *Popul Health Manag.* 2017.

Modes M, Davis A, Farnan J, Arora V. Ordering Wisely: Engaging Faculty to Champion High-Value Care Initiatives. *Am J Med Qual.* 2016;31(4):380-381.

Parikh RB, Kakad M, Bates DW. Integrating Predictive Analytics Into High-Value Care: The Dawn of Precision Delivery. *JAMA*. 2016;315(7):651-652.

Patel, M. S., et al. (2012). "The VALUE Framework: training residents to provide value-based care for their patients." Journal of General Internal Medicine 27(9): 1210-1214.Porter ME. What is value in health care? *NEJM.* 2010;363(26):2477-2481.

Porter ME. What is value in health care? *NEJM*. 2010;363(26):2477-2481.

Porter ME, Teisberg EO. *Redefining health care: creating value-based competition on results.* Boston: Harvard Business School Press, 2006.

Porter ME, Teisberg EO. How physicians can change the future of health care. *JAMA*. 2007;297(10):1103-1111.

Salmon RB, Sanderson MI, Walters BA, Kennedy K, Flores RC, Muney AM. A collaborative accountable care model in three practices showed promising early results on costs and quality of care. *Health Aff. (Millwood).* 2012;31(11):2379-2387.

Sanghavi DM, Conway PH. Paying for Prevention: A Novel Test of Medicare Value-based Payment for Cardiovascular Risk Reduction. *JAMA.* 2015;314(2):123-124.

***Value-based Payment and Related Terms***

Results: A total of 51 texts were selected to illustrate use of the term *value-based payment* and/or related terms (*value-based payment,* 22; *value-based reimbursement*, 6; *value-based purchasing*, 17; *value-based contracting*, 6).

Overview: The *representative* selections in [Table 3](#Table3) show that *value-based payment* and related terms could be used interchangeably. *Value-based contracting* appeared least frequently in the overall body of literature and has not been increasing in use since 2010 (see [Figure A-1](#FigureA1), p.20, in the Appendix). Cost and quality were mentioned as a component in almost all of the selections. Provider risk was referenced as a cost component in five articles. Almost half of the selections described benefits in terms of health outcomes but did not use the word *benefit*. Other potential definition components appeared less frequently: harms, patient experience (or consumer responsibility), populations.

**Table 3. Examples of Explicit or Implied Definitions of *Value-based Payment, Value-based Purchasing, Value-based Reimbursement* and *Value-based Contracting***

| **First Author** | **Explicit or Implied Definition** | **Perspective** |
| --- | --- | --- |
| **Value-based payment** | | |
| **Moore**  **2012** | Is your governing board ready for an increasing share of revenues coming from value-based payment? . . . They [CEOs and CFOs] see a movement away from fee for- service, and a movement toward rewarding health networks of providers for higher quality and lower total costs per capita. | Consulting firm |
| **Chien**  **2013** | The PVBM [physician value-based payment modifier, CMS] seeks to financially reward physicians who provide health care that is high value — both high in quality and low in cost. | Medicine/health policy/public health (academic) |
| **Conrad**  **2013** | . . . value-based payment, which links providers' reimbursement to the value, rather than the volume, of services delivered. With funding from the Robert Wood Johnson Foundation, eight grantees across the country are designing and implementing value-based payment reform projects. | Health services research (academic) |
| **Redding 2013** | To address this challenge, both commercial and governmental payers have begun to explore value-based payment methodologies aimed at reducing risk and waste by promoting increased coordination of healthcare services. | Consulting group |
| **Bunkers 2014** | . . . value-based payment models. The new model, which was implemented this past January, incorporates quality metrics, provides physicians with regular reports of their performance, and already has resulted in greater physician attention to outcomes, safety, and patient experience. . . . This new and evolving payment model regards value (defined as better outcomes and patient experience at a lower cost). Current productivity-based physician compensation models reward only volume of care, which can conflict with the goal of increasing value. | Large multispecialty clinic (Mayo) |
| **Ryan**  **2014** | The ideal in value-based payment is to create a single set of performance measures that spans care settings for which a single group of providers shares accountability for the health of a population. | Academic (medical school) |
| **No Authors**  **2015** | Health systems and physicians are increasingly moving toward value-based payment models. Although the goal of alignment toward the “triple aim” of improved patient outcomes, better experience and lower cost of care resonates with most, it is clear that this transition requires expertise that many hospitals and physicians groups need. | Health systems |
| **Combes**  **2015** | . . . governing boards are wrestling with is how to successfully transition to value based models of care delivery and payment. Many organizations are not yet ready to have their payments tied to achieving cost and quality objectives for patient populations and assuming some level of risk. | Health systems |
| **Kennedy 2015** | Existing value-based payment models have emphasized quality of care, clinical outcomes, patient safety, service quality, and costs. | Large family practice group (Mayo Clinic) |
| **Kotzbauer 2015** | Proponents of value-based payment models believe such models—which tie payment to predefined quality and cost-of-care goals—are critical to overcoming the inconsistent quality, unsustainable costs and fragmented care that define the U.S. healthcare system. | Health system leaders |
| **Siegel**  **2015** | Value-based payments, which reward providers for the quality and efficiency of care, as opposed to the volume of patients treated, are slowly and steadily gaining a foothold through new care models such as accountable care organizations (ACOs) and patient-centered medical homes (PCMHs). . . . But the full promise of population health management will be realized only if payers and providers fully embrace value-based payment – reimbursement that approximates, as closely as possible, the true costs of delivering proactive, comprehensive care. | Payers |
| **Stein**  **2015** | VBP [value-based payment] provides financial incentives for hospitals based on the patient experience, the premise being that patient experience is a key component of quality of care. | Hospital |
| **Wagner**  **2015** | Although fee-for-service contracts still dominate the healthcare payment landscape, the progression toward value-based payment models—which reward providers that achieve quality and cost targets—is picking up speed. . . . Shortly afterward, a group of large health systems and insurers formed a task force aimed at shifting 75 percent of their business into value-based contracts that offer incentives for meeting targets related to health outcomes, quality of care and cost management.  *NOTE: Targets in different arrangements included process, health outcomes, and/or utilization measures.* | Private payers and health systems |
| **Chaiyachati 2016** | Value-based payment models should incorporate financial support for at-risk hospitals implementing strategies to address social determinants because of their influence on quality-of-care outcomes. | Health policy (academic, clinical scholars program) |
| **Ellner**  **2017** | Design based on these principles should lead to higher-value healthcare, but will require new approaches to workforce training. . . . Payment must support the primary care functions and reward value, facilitating a paradigm shift away from visit-based healthcare. | Physician (academic) |
| **Robertson-Cooper**  **2017** | Physicians will be increasingly required to move away from fee-for-service reimbursement to a value based system that ties payment to quality and outcomes. The Affordable Care Act, the US Department of Health and Human Services Centers for Medicare & Medicaid Services (CMS) value-based payment goals, and the Medicare Access and Children’s Health Insurance Program Reauthorization Act have accelerated the move of the health care system away from a volume-based system to one that focuses on population health, care coordination, and health outcomes. | Family physicians (AAFP) and private payer |
| **Value-based purchasing** | | |
| **Cutler**  **2010** | The third category of cost-cutting measures in the law is value-based purchasing, or pay-for-performance. The law calls for linking existing measures of inpatient quality to payment, and expanding performance-based measurement and payment systems from inpatient care to outpatient care, physician services, home health care, and skilled nursing facilities. Many of the quality measures being considered are associated with lower costs—for example, improvements in patient safety. . . . The true measure of health care reform's success is whether it drives down medical costs over the long term. Providers who are paid for value, not volume, will have incentives to limit unnecessary care and will also ensure that people receive appropriate chronic and preventive care. | Health Economics (academic) |
| **Thomas 2012** | Value-based purchasing offers a set of tools to achieve 2 important goals:  • It pays providers for what consumers want the healthcare system to do, i.e., reward value and quality.  • It engages consumers to be sophisticated and knowledgeable users of healthcare and therefore encourages them to do more to improve their health."  Purchasers should reward the health plans that deliver on better quality and lower costs. | NCQA |
| **VanLare 2012** | VBP rewards providers who deliver better outcomes in health and health care for the beneficiaries and communities they serve at lower cost. | Government (CMS) |
| **Berman 2013** | Value-based purchasing (VBP) is a strategy that links payments to value of care over the entire continuum of patient treatment and hinges on recognizing and rewarding shared accountability among providers. | Physician (academic practice group) |
| **Joynt 2017** | First, whereas value-based purchasing programs reward achievement of high quality and good outcomes among all beneficiaries, we should also consider creating additional targeted financial incentives to reward achievement or improvement specifically for socially at-risk beneficiaries. | Public health (academic) |
| **Value-based reimbursement** | | |
| **Evans 2012** | Value-based reimbursement represents a fundamental shift in the way health plans pay providers for care. Rather than creating incentives for providers to deliver high-quantity care—a downside of the fee-for-service (FFS) model—value based reimbursement aims at creating incentives for providers to achieve high-quality care. Because value-based reimbursement is focused on driving healthcare value, improved population-based outcomes are an expected result. | Consulting firm |
| **Value-based contracting** | | |
| **Mehr 2013** | “Outcomes based contracting” represents the next evolution, encompassing risk-based agreements with performance guarantees or payment scales for attaining not utilization or market share benchmarks, but clinical status improvement or prevention of adverse events. This is truly value-based contracting. | Managed care |
| **Eggbeer 2015** | Most value-based contracting is based on three premises:   - Reducing cost by eliminating inappropriate utilization and lowering prices - Sharing savings or capturing a portion of the value created through lower costs - Increasing volume through market share gains resulting from enhanced value to the customer   Value-based contracting starts with understanding the customer’s view of value. In the emerging retail healthcare market for many healthcare services and insurance products, value equates with price, which is becoming a primary factor in consumer decisions (i.e., purchase decisions made directly by consumers, rather than on their behalf by employers, payers, or providers). . . . Price, however, is not the only criterion for describing value. Other important factors are access, choice, patient experience, pricing transparency, and premium cost predictability— all of which are key considerations in the design of value-based contracts. | Large healthcare systems and private payers |

References for Definitions of *Value-based Payment* and Related Terms

No Authors. Value-based Payment Readiness. *Healthc Financ Manage.* 2015;69(7):suppl 1-4.

Berman B, Pracilio VP, Crawford A, et al. Implementing the physician quality reporting system in an academic multispecialty group practice: lessons learned and policy implications. *Am J Med Qual.* 2013;28(6):464-471.

Bunkers B, Koch M, McDonough B, Whited B. Aligning physician compensation with strategic goals. *Healthc. Financ. Manage.* 2014;68(7):38-45.

Chaiyachati KH, Grande DT, Aysola J. Health systems tackling social determinants of health: promises, pitfalls, and opportunities of current policies. *Am J Manag Care.* 2016;22(11):e393-e394.

Chien AT, Rosenthal MB. Medicare's physician value-based payment modifier--will the tectonic shift create waves? *N. Engl. J. Med.* 2013;369(22):2076-2078.

Combes JR, Totten MK. Leadership for value-based care. Four areas of knowledge and skills must be mastered to make the move from fee for service. *Healthc Exec* 2015;30(3):74, 76-77.

Conrad D, Grembowski D, Gibbons C, et al. A report on eight early-stage state and regional projects testing value-based payment. *Health Aff (Millwood).* 2013;32(5):998-1006.

Cutler D. Analysis & commentary. How health care reform must bend the cost curve. Health Aff. (Millwood). 2010;29(6):1131-1135.Eggbeer B, Sears K, Homer K. Finding the 'sweet spot' in value-based contracts. *Healthc Financ Manage.* 2015;69(8):48-57.

Eggbeer B, Sears K, Homer K. Finding the 'sweet spot' in value-based contracts. *Healthc Financ Manage.* 2015;69(8):48-57.

Ellner AL, Phillips RS. The Coming Primary Care Revolution. *J Gen Intern Med*. 2017;32(4):380-386.

Evans J. The policy-driven health plan: a road map for value-based reimbursement. *Am Health Drug Benefits.* 2012;5(7):446-450.

Joynt KE, De Lew N, Sheingold SH, Conway PH, Goodrich K, Epstein AM. Should Medicare Value-Based Purchasing Take Social Risk into Account? *NEJM.* 2017;376(6):510-513.

Kennedy DM, Nordrum JT, Edwards FD, Caselli RJ, Berry LL. Improving service quality in primary care. *Am J Med Qual.* 2015;30(1):45-51.

Kotzbauer G, Weeks WB. Paving the road to success under value-based payment models. *Healthc Financ Manage.* 2015;69(1):84-85.

Mehr SR. Value-based contracting for pharmaceuticals: getting ready for prime time? *Am J Manag Care.* 2013;19 Spec No. 3:E1.

Moore KD, Eyestone K, Coddington DC. Governing in an era of value-based payment. *Healthc Financ Manage.* 2012;66(12):100-102.

Redding J. Achieving clinical integration. *Healthc Financ Manage.* 2013;67(11):56-58, 60.

Robertson-Cooper H, Neaderhiser B, Happe LE, Beveridge RA. Family Physician Readiness for Value-based Payments: Does Ownership Status Matter? *Popul Health Manag.* 2017.

Ryan AM, Press MJ. Value-based payment for physicians in Medicare: small step or giant leap? *Ann. Intern. Med.* 2014;160(8):565-566.

Siegel M. Risk-adjusted base payments can support the move to value. *Healthc Financ Manage*. 2015;69(1):38-41.

Stein SM, Day M, Karia R, Hutzler L, Bosco JA, 3rd. Patients' perceptions of care are associated with quality of hospital care: a survey of 4605 hospitals. *Am J Med Qual.* 2015;30(4):382-388.

Thomas S, O'Kane M. Value-based purchasing. *Am J Manag Care.* 2012;18(11):750-752.

VanLare JM, Conway PH. Value-based purchasing--national programs to move from volume to value. *NEJM.* 2012;367(4):292-295.

Wagner K. Shifting Gears: The Move Toward Value-based Payment. *Healthc Exec.* 2015;30(3):10-12, 14-16, 18.

***Definition Components***

The following table shows some of the detail that was used in the 59 selected articles to describe the pre-identified potential components of definitions. Texts were considered to refer to benefits if they mentioned clinical or health-related outcomes and were considered to refer to quality if they referred to care processes or used the term *quality* without supporting detail.

**Table 4. Specific Language Related to Potential Definition Components (*Value-based Care, Value-based Payment,* and Related Terms)**

| **Definition Components** | ***Examples of specifics where mentioned*** |
| --- | --- |
| Cost | *efficiency, cost-effectiveness, smarter spending, lower cost, risk, overall cost savings, unsustainable, waste, lower prices* |
| Contrasted with Volume | *contrasted with fee-for-service, “fee-for-value” versus “fee-for-volume”, visit-based healthcare* |
| Benefits | *prevention, health* *outcomes, clinical outcomes, social determinants of health, population-based outcomes, healthier people, contrast with process measures* |
| Harms | *patient safety, safety indicators, reduction in adverse events* |
| Quality | *performance measures, performance guarantees tied to outcomes, provider performance, proactive comprehensive care, targets, care delivery, accessibility, coordinated care, [non]fragmented care, better care, process of care* |
| Patient Experience | *goals set jointly by patient and provider, service quality, customer’s (consumer’s) view of value, pricing transparency, premium predictability, choice; engages consumers to be sophisticated and knowledgeable users of healthcare and therefore encourages them to do more to improve their health* |
| Defined Population | *population served by a provider group or affiliation, patient panel, patients across care settings for whom a single group of providers is accountable, communities served by providers, plan population, socially at-risk population* |
| Other | *“Triple Aim”, innovations, increasing volume through market share gains, transparency/accountability* |

***Other Findings***

See the Appendix for information on the frequency of use of terms over time and use by different stakeholders ([Figure A-2](#FigureA2), p. 21).

## Appendix: *Value-based Care, Value-based Payment* and Related Terms

**Table A-1. Journals Included in the Review**

| 1. American Family Physician 2. American Health Drug Benefits 3. American Journal of Health Systems Pharmacy 4. American Journal of Managed Care 5. American Journal of Medical Quality 6. American Journal of Preventive Medicine 7. American Journal of Public Health 8. Annals of Family Medicine 9. Annals of Internal Medicine 10. Health Affairs 11. Healthcare Executive 12. Healthcare Financial Management 13. Journal of American Board of Family Medicine | 1. JAMA 2. JAMA Internal Medicine 3. Journal of General Internal Medicine 4. Journal of Healthcare Management 5. Journal of Medical Practice Management 6. Journal of the American Geriatrics Society 7. Journal of the American Medical Directors Association 8. Journal of Managed Care Pharmacy 9. NEJM 10. Obstetrics and Gynecology 11. Population Health Management |
| --- | --- |

**Figure A-1. Number of Articles with Terms Related to *Value-based Care* or *Value-based Payment* in Title or Abstract, 2010-2016, Any Journal**

**Year of Publication**

**Number of Articles**

**Use of Terms by Different Stakeholders**

In Figure A-2, *perspective* refers to the perspective of the authors, typically derived from the principal author’s affiliation as identified in the article. The distribution for *high-value care* is included to show the contrast in stakeholder usage between *high-value care* and the terms of interest*.* Eight of the 12 articles using the term *high-value care* were written by physicians representing the American College of Physicians.

**Figure A-2. Mix of Author Perspectives in Articles Selected for Definitions/Uses of *Value-based Care, Value-based Payment* and Related Terms**

# Population Health and Population Medicine

## Statement of Goals – Pop Health/Pop Medicine Review

To identify the definitions *population health* and *population medicine* in the recent literature (2010 to 2017), elucidate the elements incorporated in those definitions, and describe the perspectives (primary affiliation) of the lead authors of the literature cited.

## Methods – Pop Health/Pop Medicine Review

PubMed was searched from 1/1/2010 to 12/1/2017 and a scan of the grey literature through a search of organizational websites referenced in the published literature was performed. Article selection was limited to English language and for *population health* to 27 journals (See [Table A-1](#TableA1), Appendix, p. 20). For *population medicine*, no journal restrictions were used and search dates were from 1/1/2010 to 12/14/2017. Exclusion criteria upon review included: too narrow a clinical focus, non-US perspective, focus on purchase of drugs or devices, non-human subject focus, and not generally relevant to topic. The abstract and full-text of each article were examined for any explicit definitions or illustrative uses of the terms of interest. Dual review of the abstracted articles and definitions was performed.

## Results – Pop Health/Pop Medicine Review

### Definitions on Organization Websites

Definitions ascertained from the grey literature of organizational websites (referenced in the reviewed literature) for the terms *population health*, *population medicine* and closely related terms are provided ([Table 5](#Table5)). These definitions may be useful to refer to in the panel discussions.

Additional information presented in this report includes a framework for population health by Kindig ([Figure 2](#Figure2), p. 25) and the perspectives of the authors of definitions provided for *population health* and *population medicine* ([Figure A-3](#FigureA3), p. 37).

**Table 5: Definitions Identified on Organizational Websites, *Population Health*  and *Population Medicine***

| **Organization/Blog** | **Definition** |
| --- | --- |
| **Improving population health: Policy, Practice, Research.**  Editor: David A. Kindig, MD, Ph.D. Access date: 1/14/2018 | **Population health** is defined as the health outcomes of a group of individuals, including the distribution of such outcomes within the group.  These groups are often geographic populations such as nations or communities, but can also be other groups such as employees, ethnic groups, disabled persons, prisoners, or any other defined group. The health outcomes of such groups are of relevance to policy makers in both the public and private sectors. <http://www.improvingpopulationhealth.org/blog/what-is-population-health.html>  [Figure 2](#Figure2) (Framework) (p. 25) |
| **Institute for Healthcare Improvement (IHI)**  Access date: 1/14/2018 | **Population Medicine** is the design, delivery, coordination, and payment of high-quality healthcare services to manage the Triple Aim for a population using the best resources we have available within the healthcare system.  Much of the efforts today such as the ACO, risk stratification methods, patient registries, Patient Centered Medical Home, and other models of team-based care are all part of a comprehensive approach to population medicine.  **Population Management:** The term population management should be clearly distinguished from population health (which focuses on the broader determinants of health). From what we have seen through our work at IHI, population management as presently practiced is best conceptualized as *population medicine.*  [*http://www.ihi.org/communities/blogs/population-health-population-management-terminology-in-us-health-care*](http://www.ihi.org/communities/blogs/population-health-population-management-terminology-in-us-health-care) |
| **Institute for Healthcare Improvement (IHI)**  Access date: 1/14/2018 | **Triple Aim:** The IHI Triple Aim is a framework for optimizing health system performance by simultaneously accomplishing three critical objectives, which we call the Triple Aim: improve the health of the population; enhance the patient experience of care (including quality, access, and reliability); and reduce, or at least control, the per capita cost of care. <http://www.ihi.org/sites/search/pages/results.aspx?k=triple+aim>  The Triple Aim is an ongoing IHI learning initiative to better understand new models that can improve the individual patient experience and the health of entire communities, at a reasonable cost. <http://www.ihi.org/sites/search/pages/results.aspx?k=population+health> |
| **World Health Organization (WHO)**  Access date: 1/14/2018 | **Public Health** is an organized effort by society, primarily through its public institutions to improve, promote, protect, and restore the health of the population through collective action. It includes services such as health situation analysis, health surveillance, health promotion, prevention, infectious disease control, environmental protection and sanitation, disaster and health emergency preparedness and response, and occupational health, among others. <http://www.who.int/healthsystems/hss_glossary/en/index8.html> |
| **Agency for Healthcare Research and Quality, National Quality Measures Clearinghouse (NQMC)** | **Population Health Measures:** measures that address health issues of individuals or populations defined by residence in a geographic area or a relationship to organizations that are not primarily organized to deliver or pay for health care services (such as schools or prisons). The responsibility for “performance” typically falls to public officials, public health agencies, or organizations that are not primarily deliverers of care. [www.qualitymeasures.ahrq.gov/about/domain-frame work.aspx](http://www.qualitymeasures.ahrq.gov/about/domain-frame%20work.aspx) |
| **National Quality Forum Report: Improving Population Health by Working with Communities-Action Guide 1.0, 8/1/2014**  Accessed 1/15/2018 | **Population health**-The health of a population, including the distribution of health outcomes and disparities in the population (Kindig, 2003)  **Population**-All individuals in a specified geopolitical area  **Sub-population**-A group of individuals that is a smaller part of a population. Subpopulations can be defined by geographic proximity, age, race, ethnicity, occupations, schools, health conditions, disabilities, interests, or any number of other shared characteristics.  **Health** – A state of complete physical, mental, and social well-being and not merely the absence of disease or infirmity. (WHO preamble, 1946)  **Determinants of Health** – Factors affecting the health of individuals in a population or subpopulation, such as the social and physical environment, behaviors, and healthcare. <http://www.who.int/hia/evidence/doh/en/>  **Health Disparities** – Difference in health status or health outcomes within a population (IOM, 2002, Unequal Treatment: Confronting Racial and Ethnic Disparities in Health Care).  **Health Equity**-– The absence of systmat6ic disparities in health or major social determinants of health between groups with different underlying social or economic advantages.  **Health Inequity** – Difference in health status between groups with varying social and economic advantage/disadvantage (e.g., socioeconomic status, gender, age, physical disability, sexual orientation and gender identity, race and ethnicity) that are caused by inequitable, systemic differences in social conditions (i.e., policies and circumstances that contribute to health determinants).  <http://www.qualityforum.org/Project_Pages/Population_Health_Framework.aspx> |
| **Centers for Medicare & Medicaid Services; Centers for Medicare & Medicaid Innovation. Suggested population level measures for the CMS State Innovation Model Initiative**  Accessed 1/15/2018 | We define **population health** as the factors that influence the health outcomes of groups of individuals, including the distribution and equity of such outcomes across various segments of society (adopted from Kindig et al, Am J Public Health, 2003,380-383)  <https://innovation.cms.gov/files/x/SIMPopHlthMetrics.pdf> |
| **CDC Community health assessment for population health improvement**  Accessed 1/15/18 | Uses **framework for population health planning** from Kindig DA, 2008. JAMA. 299(17), 2081-2083.  <https://wwwn.cdc.gov/communityhealth/pdf/final_chaforphi_508.pdf> |
| **Population Health Alliance**  Access 1/15/18 | **Population Health Management**: A population health management program strives to address health needs at all points along the continuum of health and well-being through participation of, engagement with and targeted interventions for the population.  <http://www.populationhealthalliance.org/research/understanding-population-health.html> |

**Figure 2: Framework for Population Health (Kindig)**


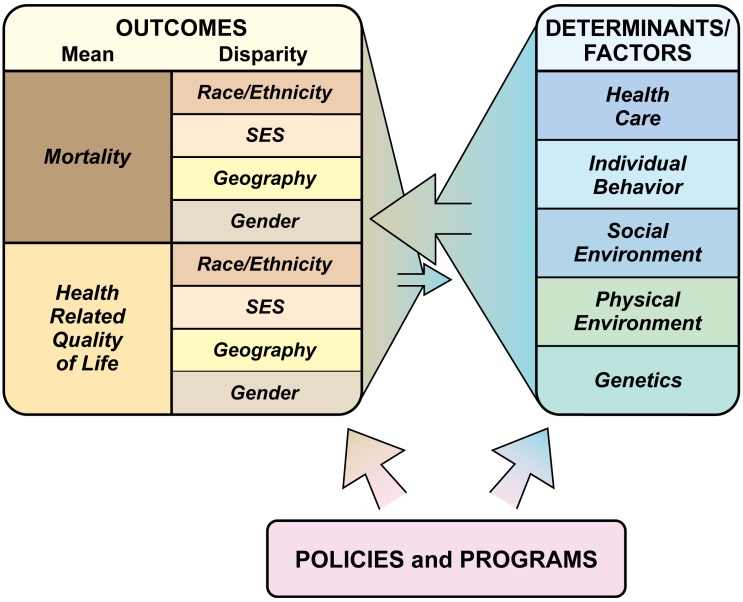


### Literature Search Results

In the review of *population health* 603 articles were originally identified. Of these, 118 were identified as having a definition or providing meaningful context for the construct: 35 had an explicit or implicit definition of *population health*, 8 additional had a definition of population health management, and 75 provided context for the construct but no specific definition (not included in this report). If the definition identified was a previously published definition, the original author and manuscript were included in the tables. [Table 6](#Table6) (p.27) presents 18 definitions of *population health* and [Table 7](#Table7) (p. 31) illustrates the components of those definitions. Thirty-four (34) articles were identified with the term *population medicine* in the title or abstract and within the date range. Of these, five were found to have definitions of *population medicine* ([Table 8](#Table8), p. 32); definition components are shown in [Table 9](#Table9) (p. 34). The term *population health management* was not searched explicitly but definitions that emerged from the search of the term *population health* were included in this report for reference. These 10 definitions of *population health management* are presented in a table ([Table 10](#Table10), p. 34).

### Definitions of Population Health and Population Medicine

***Population Health***

Results: Eighteen (18) definitions of *population health* were identified from the literature. If a definition was used in an article in the scoping review but referenced a previous definition, the original author is listed in the table below.

Overview: Several articles referenced the definition put forth by Dr. Kindig in a 2003 article from the *American Journal of Public Health*. Most definitions included a reference to the determinants of health, the importance of defining the population, and outcomes of health. Some definitions also described the domains of health. Abstracted definitions, the perspective or affiliation of the first author, and the year of publication and reference are presented in [Table 6](#Table6) (p. 27), which is followed by a table that identifies components of the definitions ([Table 7](#Table7), p. 31).

**Table 6: Population Health Definitions and Perspectives**

| Author | Definition | Perspective |
| --- | --- | --- |
| Dunn 1999 | The health of a population as measured by health status indicators and as influenced by social, economic and physical environments, personal health practices, individual capacity and coping skills, human biology, early childhood development, and health services. | Public Health & Policy Academia |
| Gourevitch 2012 | Health of a group of individuals, which public health agencies define by geography and health delivery systems define by people receiving care (such as all the patients in a particular accountable care organization). | Public Health & Policy Academia |
| Kindig 2003 | The health outcomes of a group of individuals, including the distribution of such outcomes within the group… the field of population health includes health outcomes, patterns of health determinants, and policies and interventions that link these two… These populations are often geographic regions, such as nations or communities, but they can also be other groups, such as employees, ethnic group’s disabled persons, or prisoners.” PH refers to health “over a period of years” rather than “a point in time.” | Public Health & Policy Academia |
| Hacker 2013 | Population health is seen in two distinct ways: (1) from a public health perspective, populations are defined by geography of a community (e.g., city, county, region, state, or national levels) and (2) from the perspective of the delivery system (individual providers, groups of providers, insurers, and health delivery systems), population health denotes a ‘panel’ of patients served by the organization. | Community Health Institute |
| Noble 2013 | ACOs interpret population health as a “responsibility to provide preventive care for all their patients and care management for their patients with serious chronic diseases… “Population health depends not only on medical care, but also on social services, the public health system, and crucially, on socioeconomic factors (e.g., housing, education, poverty, and nutrition). | Public Health & Policy Academia |
| Sanford 2013 | Improving health outcomes in whole populations through health promotion and disease prevention activities. | Academic Medical Center |
| Sharfstein 2014 | A term that includes the “underlying causes of illness” beyond the “biomedical paradigm,” including “the impact of income inequality, educational differences, and unjust disparities.” | State Department of Health |
| Nash 2015 | Includes academic components such as the social determinants of health as well as pragmatic components such as improvement of many health metrics and appropriate management of economic risks for clinical decision making. | Public Health & Policy Academia |
| Diez Roux 2016 | A conceptual approach to understanding health… that has two key principles: (1) The need to consider factors defined at multiple levels of organization in understanding health and acting to improve it (this implies integrating social and biologic processes), and (2) an explicit concern with health equity because we cannot substantially improve the health of the population as a whole without addressing health inequities and because drivers of health inequities are often the drivers of the health of the population generally. | Public Health & Policy Academia |
| Keyes 2016 | Population health science is the study of the conditions that shape distributions of health within and across populations, and of the mechanisms through which these conditions manifest as the health of individuals. | Public Health & Policy Academia |
| Arora 2016 | Population well-being – a measure that incorporates physical, emotional, and social health – may help explain variation in life expectancy. Well-being includes not only the absence of disease but also a sense of opportunity, happiness and lack of stress. It reflects the ability to afford food, housing, and health care; to live in a safe neighborhood; and to work in a trusting, respectful environment. In other words, well-being comprehensively assesses both individual- and neighborhood-level characteristics that may influence the health and longevity of an entire community. | Public Health & Policy Academia |
| Casarett 2016 | Focuses on improving the health of populations, with a special emphasis on reducing disparities in health outcomes and improving the value of healthcare. | Academic Medicine |
| Baehr 2016 | Despite this widespread policy focus on population health, a common definition of population remains elusive. To insurers, for example, it is a group of beneficiaries, while to health care providers it is a patient panel. Hospitals focus on inpatients while both policy makers and public health officials consider all of the people living in their jurisdiction. Other candidate units for describing total population health (TPH) include ACOs, hospital referral regions, or a rational service area, such as a medically underserved area. | US Department of HSS |
| Harris 2016 | Two different approaches to PH exist: a broad view in with PH delivers “improved outcomes by tackling large-scale social, economic, and environmental issues”, using “public health tools and techniques to create conditions to promote health, prevent disease, and ensure patients recover after medical treatment” and in which “individuals are not identified and may not be attributed to any particular health care provider”. In contrast, a narrow definition of PH includes “activities that address the specific health needs of discretely defined, enumerated, and engaged patient subpopulations under the scope and care of a particular health care system or clinical team”; patients are known “to a particular care team” or provider. | Public Health & Policy Academia |
| Kassler 2017 | The health of a population (including mortality, quality of life, and functional status) as determined by access to services, quality of care, health behavior, social environment, and the physical environment. | Centers for Medicare and Medicaid Services US Gov. |
| Pimperl 2017 | The health of the population of insured persons attributed to the ACO by contract. | Public Health & Policy Academia |
| May 2017 | Health outcomes of a group of individuals, including the distribution of such outcomes within the group. The groups are often geographic populations such as nations or communities, but also can be other groups such as patient panels, employees, health plan members, ethnic groups, or any other defined group. Medical care is only one of many factors that affect population health interventions (e.g., immunizations, fluoridations), the social environment and policies (e.g., education, employment, social support), the physical environment (e.g., urban design, clean air, water), genetics, individual lifestyle and behavior (e.g., diet, physical activity) and stress (e.g., depression, racism). | Institute of Biotechnology, Academia |
| Allen 2017 | The distribution of health outcomes in a population and the health determinants that influence this distribution. | Medicare Provider/Contractor |

**Key: PH**: Population health; **CPM**: Clinical population medicine; **ACO**: Accountable care organization; **PWB**: Population Well-Being; **TPH**: Total population health; **ACOs**: Accountable care organizations. A rational service area is a discrete geographic unit that represents potential primary care utilization based on travel time to various health services. The medically underserved area designation makes an area eligible for additional federal funding and is based on an insufficient number of primary care providers, high poverty rate, and/or high rates of infant mortality.^13^

References for Population Health Definitions

Allen H, Burton WN, Fabius R. Value-Driven population health: An Emerging Focus for Improving Stakeholder Role Performance. *Popul Health Manag.* 2017;20(6):465-474.

Arora A, Spatz E, Herrin J, et al. Population Well-Being Measures Help Explain Geographic Disparities In Life Expectancy At The County Level. *Health Aff (Millwood).* 2016;35(11):2075-2082.

Baehr A, Holland T, Biala K, Margolis GS, Wiebe DJ, Carr BG. Describing Total population health: A Review and Critique of Existing Units. *Popul Health Manag.* 2016;19(5):306-314.

Casarett D, Teno J. Why population health and Palliative Care Need Each Other. *JAMA.* 2016;316(1):27-28.

Dunn JR, Hayes MV. Toward a lexicon of population health. *Can J Public Health.* 1999;90 Suppl 1:S7-10.

Gourevitch MN, Cannell T, Boufford JI, Summers C. The challenge of attribution: responsibility for population health in the context of accountable care. *American journal of preventive medicine.* 2012;42(6 Suppl 2):S180-183.

Hacker K, Walker DK. Achieving population health in accountable care organizations. *American journal of public health.* 2013;103(7):1163-1167.

Harris D, Puskarz K, Golab C. population health: Curriculum Framework for an Emerging Discipline. *Popul Health Manag.* 2016;19(1):39-45.

Kassler WJ, Howerton M, Thompson A, Cope E, Alley DE, Sanghavi D. population health Measurement at Centers for Medicare & Medicaid Services: Bridging the Gap Between Public Health and Clinical Quality. *Popul Health Manag.* 2017;20(3):173-180.

Keyes K GS. *population health Science.* New York, NY: Oxford University Press; 2016.

Kindig D, Stoddart G. What is population health? *American journal of public health.* 2003;93(3):380-383.

May T, Byonanebye J, Meurer J. The Ethics of population health Management: Collapsing the Traditional Boundary Between Patient Care and Public Health. *Popul Health Manag.* 2017;20(3):167-169.

Nash DB. Population health: where's the beef? *Popul Health Manag.* 2015;18(1):1-3.

Noble DJ, Casalino LP. Can accountable care organizations improve population health?: should they try? *JAMA.* 2013;309(11):1119-1120.

Orkin AM, Bharmal A, Cram J, Kouyoumdjian FG, Pinto AD, Upshur R. Clinical Population Medicine: Integrating Clinical Medicine and population health in Practice. *Annals of family medicine.* 2017;15(5):405-409.

Pimperl A, Schulte T, Muhlbacher A, et al. Evaluating the Impact of an Accountable Care Organization on population health: The Quasi-Experimental Design of the German Gesundes Kinzigtal. *Popul Health Manag.* 2017;20(3):239-248.

Roux AV. On the Distinction--or Lack of Distinction--Between population health and Public Health. *American journal of public health.* 2016;106(4):619-620.

Sanford ED, Handley MA. Population health and accountable care organizations. *JAMA.* 2013;310(3):323-324.

Sharfstein JM. The strange journey of population health. *Milbank Q.* 2014;92(4):640-643.

**Table 7: *Population Health* Definition Components and Examples**

| Definition Elements | Examples |
| --- | --- |
| Social Determinants of Population Health | *Social: housing, education, transportation, nutrition*  *Economic: poverty, income, inequality, disparities*  *Physical Environment* |
| Individual Determinants | *Personal health behavior; Individual capacity and coping skills, stress*  *Human biology and genetics, Early childhood development* |
| Health Policy and Public Health | *Health services, Access, Social services*  *Public health system, Health promotion activities*  *Disease prevention activities*  *Concern with health equity* |
| System Determinants | *Management of economic risks for clinical decision making*  *Quality of Care* |
| Definition of Population | *Group of Individuals*  *Geography (nations or communities)*  *Health delivery systems (individual providers, groups of providers, insurers, group of beneficiaries) Panel of patients of provider or organization (ACO)*  *Hospital referral regions*  *Medically underserved area Employees, Covered by given insurance program Group characteristics: ethnicity/race, disabled, medically complex conditions* |
| Describing “health” | *Mortality, Quality of life, Functional status*  *Physical, emotional, and social health*  *Well-being assesses both individual and neighborhood level characteristics* |
| Outcomes and Measures | *Health status indicators Health over a period of years Value of health care*  *Reducing disparities*  *Patient and provider level data as indicators for clinical decisions; system designs responsive to changing community needs* |
| Statistics/Causal Pathways | *Patterns of health determinants*  *Distributions of health within and across populations*  *Mechanism through which conditions impact health* |

***Population Medicine***

Results: Of the 34 identified articles, five were found to have relevant definitions, as shown in Table 8. An additional organizational website was identified.

Overview: There were a limited number of references that put forth definitions of *population medicine*. In general, the writers agreed that population medicine was the practice of principles of population health in the context of medical care delivery. The term was also used in the context of medical education programs that sought to teach the principles of population health and practice of population medicine. [Table 9](#Table9) (p. 34) presents components of the definitions and examples of the language used.

**Table 8: *Population Medicine* Definitions and Perspectives**

| Author | Definition | Perspective |
| --- | --- | --- |
| Kindig 2012 | References the Department of Population Medicine at Harvard Pilgrim Health Care Institute as defining PM as “The specific activities of the medical care system that, by themselves or in collaboration with partners, promote population health beyond the goals of care of the individuals treated”. [www.improvingpopulationhealth.org/blog/2012/06](http://www.improvingpopulationhealth.org/blog/2012/06) | Public Health & Policy Academia |
| Gray 2013 | The new responsibilities for the clinician practicing population medicine not only include maximizing value by getting the right outcomes for the right patients in the right place with the least use of resources, but also ensuring the prevention of inequity related to age or gender or race or social class. | Academic Medicine NHS |
| White 2013 | Population medicine, a term developed by the Institute for Healthcare Improvement (IHI), refers to healthcare services designed to care for populations of people. Specifically, population medicine aims to improve the patient experience of care (both quality and satisfaction), improve the health of populations, and reduce healthcare associated costs. These three goals together define the Triple Aim, an IHI initiative to optimize health system performance. Population medicine differs from public health and population health in its focus on system design to improve outcomes.  PM is the design, delivery, coordination, and payment of high-quality healthcare services to manage the Triple Aim for a population using the best resources we have available within the healthcare system.  Much of the efforts today such as the ACO, risk stratification methods, patient registries, Patient Centered Medical Home, and other models of team-based care are all part of a comprehensive approach to population medicine. | Academic Medicine  (IHI website reference) |
| Orkin 2017 | Clinical Population Medicine (CPM) is a conscientious, explicit, and judicious application of population health approaches to care for individual patients and design health care systems. CPM integrates clinical care and **community** health by engaging with both patients and populations simultaneously. CPM practitioners are integrators from any existing field of practice, who consider and deliver every aspect of their care for the mutual benefit of individual patients and the prevention and treatment of illness in the entire community.  CPM is   1. A deliberate practice applied by practitioners and institutions 2. Engaged healthcare institutions that reduce health inequity through improved access, health promotion and disease prevention 3. Using patient and population level data to deliver immediate and accessible indicators for clinical decisions and system design that are responsive to changing community health needs 4. Grounded in the science of epidemiology and medicine, and the practice of public health and clinical care | Health Care System |
| Lavigne 2017 | The role of medicine and, more broadly, healthcare in population health is known as population medicine. | School of Pharmacy |

References for *Population Medicine* Definitions

Gray JA. The shift to personalised and population medicine. Lancet 2013 Jul 20:382 (9888):200-1.

Kindig DA. (2007). [Understanding population health Terminology](http://www3.interscience.wiley.com/journal/118541248/abstract?CRETRY=1&SRETRY=0). Milbank Quarterly, 85(1), 139-161.

Kindig DA. Is Population Medicine population health? Posted on 6/13/2012 in population health Basics. [www.improvingpopulationhealth.org/blog/2012/06](http://www.improvingpopulationhealth.org/blog/2012/06)]

Kindig, DA, Stoddart G. (2003). [What is population health?](http://ajph.aphapublications.org/cgi/content/abstract/93/3/380) American Journal of Public Health, 93, 366-369.

Lavigne JE, Brown J, Matzke GR. Population health and medicine: Policy and financial drivers. Am J Health Syst Pharm. 2017 Sep 15:74(18):1413-1421.

Lewis N. Populations, population health, and the evolution of population management: making sense of the terminology in US health care today. Institute for Healthcare Improvement leadership blog.

Orkin AM, Bharmal A, Cram J, Kouyoumdjia FG, Pinto AD, Upshur R. Clinical population medicine: Integrating clinical medicine and population health in practice. Ann Fam Med. 2017 Sep:15 (5):405-409.

White J, Riese A, Clyne B, Vanvleet MW, George P. Integrating population and clinical medicine: A new third-year curriculum to prepare medical students for the care of individuals, panels, and populations. RI Med, 2015, Vol. 98(9), p32-25.

**Table 9: *Population Medicine* Definition Components and Examples**

| Components | Examples |
| --- | --- |
| Application of population health principles | *Integration of clinical care and community health*  *Engagement of patients and populations*  *Treatment and prevention*  *Awareness of impact of social determinants on health* |
| Healthcare system design | *Risk stratification*  *Patient registries*  *Team-based care*  *Patient Centered Medical Home* |
| Reduce inequities | *Collaborate with partners* |
| Manage the Triple Aim (improve individual experience, improve the health of populations, reduce the per-capita costs of care for populations) | *Maximize value* |

***Population Health Management***

Results: In the scoping review of *population health,* 10 articles were identified with a definition of *population health management* and are listed below with the perspective/affiliation of the first author.

Overview: These definitions (Table 10) described a system or approach to care that focuses on health in the population as the outcome in contrast to the health of an individual who presents for care. The term *population health management* was not systematically searched, and a definition of this term is not within the scope of the Delphi panel project.

**Table 10: Definitions of *Population Health Management***

| Author | Definition | Perspective |
| --- | --- | --- |
| Matthews 2012 | A systematic approach to ensuring that all patients receive appropriate preventive, chronic, and transitional care. Enhances “the cost-effectiveness of care delivery by focusing not only on meeting the health needs of people who are sick or in immediate need of care, but also on ensuring the wellness of their entire patient population”... “Requires a healthcare provider not only to track the steps it has taken to identify and meet the healthcare needs of its patients, but also to monitor their health between visits or episodes of care so it can intervene proactively, give patients appropriate support, and engage them in their own care…Although population health management focuses partly on the high-risk patients who generate the majority of healthcare costs, it systematically addresses the preventive and chronic care needs of every patient. | Central Virginia Health Network and MedVirginia |
| Murphy 2012 | PHM has been described as “a proactive, organized, and cost-effective approach to prevention that utilizes newer technologies to help reduce morbidity while improving health status, health service use, and personal productivity of individuals in defined populations (Chapman and Pelietier, 2014). | Public Health & Policy, Academia |
| Handmaker 2015 | An approach to care aims to maximize the health of all individuals who receive their health care from the organization. | Population Health Strategies, Phytel, Dallas |
| Merahn 2015 | PHM shifts the focus from caring for patients who self-select for care to taking on transcendent responsibility for the health status of a cohort or population of patients, and has come to be associated with evolutionary trends in payment or compensation for healthcare services known variously as “accountable care” or “value-based care.” | CMO, US Medical Management |
| Washington 2016 | Using a global budget to manage the health of a specific population, generally those who seek care or may eventually seek care at a health system or institution. Geographic area, insurance enrollment, health center or health care professional group, demographic profile, similar health conditions, or other criteria may define the population. | Academic School of Medicine |
| Coe 2017 | NDQA summarizes PHM as using the “comprehensive health assessment and evidence-based decision support based on complete patient information and clinical data to manage the health of its entire population.” <http://www.ncqa.org/programs/recognition/practices/patient-centered-medical-home-pcmh#cw> | Clinical Pharmacy, Academia |
| Matthews MR 2017 | PMH is broadly defined as the advancement of the health of a defined population through coordinated programs and activities that address medical or social determinants, or both, of health and are supported by an aligned payment model that rewards improvement of population health (References the Chartis Group, Population Health Management. 2015). | Academic Medicine |
| May 2017 | PHM incorporates social determinants of health. Social factors are powerful determinants of health. Under this framework, behaviors (e.g., smoking, physical activity, nutrition, alcohol use), stress (depression and racism), socioeconomic status (educational attainment and income), genetics, access to medical care, and environment (pollution, infections, and toxins) are major determinants of health outcomes… PHM dissolves the traditional distinction of clinical medicine and public health. At root, PHM represents a fundamentally different approach to the practice of medicine in that it broadens the focus of concern for physicians beyond the single immediate patient encounter. In this approach, PHM represents a new paradigm for understanding the fiduciary duties within the physician and patient relationship. The quality domains of safety, accessibility, care coordination; effectiveness, efficiency, and equity depend on population-based approaches to care delivery. In PHM, physicians aim to maximize value, remove harms, and minimize costs for patients. | Institute for Biotechnology |
| Sandborn 2017 | An intensive focus on the overall health of a given population in an effort to improve care, reduce costs, and promote wellness. | Medical Center |
| Steenkamer 2017 | Scoping Review of Term 2000-2015: Outlines conceptualization and operationalization of PHM. Most definitions included the aim of PHI combined with of quality of care or cost containment. Moderate variation found in terms of the overall aim of PHM and PHM activities mentioned. In most definitions, populations were defined in medical terms. CMS Accountable Health Communities (AHC) aim to reduce underlying health-related social needs to reduce health care costs/utilization and improve health outcomes in community-dwelling Medicare and Medicaid beneficiaries. Few definitions included HIT or “integrator” such as ACO. Contextual factors such as health policies, legislation, markets, contracting, and readiness for change in culture. | Social Science, Academia, Netherlands |

References for *Population Health Management*

Chapman LS, Pelletier KR. Population health management as a strategy for creation of optimal healing environments in worksite and corporate settings. The Journal of Alternative and Complementary Medicine. 2004;10(S1)

Coe AB, Choe HM. Pharmacists supporting population health in patient-centered medical homes. Am J Health Syst Pharm. 2017 Sep 15 (74(18):1461-1466.

Handmaker K, Hart J. 9 steps to effective population health management. Healthc Financ Manage. 2015;69(4):70-76.

Matthews MB, Hodach R. Automation is key to managing a population's health. Healthc Financ Manage. 2012;66(4):74-78, 80.

May T, Byonanebye J, Meurer J. The Ethics of population health Management: Collapsing the Traditional Boundary Between Patient Care and Public Health. Popul Health Manag. 2017;20(3):167-169.

Merahn S. Knowledge representation and care planning for population health management. J Med Pract Manage. 2015. Sep-Oct;31(2):126-30.

Murphy SM, McGready J, Griswold ME, Sylvia ML. A method for estimating cost savings for population health management programs. Health Serv Res. 2013 Apr:48 (2Pt1): 582-602.

Sanborn MD. Population health management and the pharmacist's role. Am J Health Syst Pharm. 2017;74(18):1400-1401.

Steenkamer BM, Drewes HW, Heijink R, Baan CA, Struijs JN. Defining population health Management: A Scoping Review of the Literature. Popul Health Manag. 2017;20(1):74-85.

Washington AE, Coye MJ, Boulware LE. Academic Health Systems' Third Curve: population health Improvement. JAMA. 2016;315(5):459-460.

## Appendix: *Population Health* and *Population Medicine*

**Table A-2: Journals for *Population Health* Search**

| American Family Physician  American Health and Drug Benefits  American Journal of Health-System Pharmacy  American Journal of Medical Quality  American Journal of Preventive Medicine  American Journal of Public Health  Annals of Family Medicine  Annals of Internal Medicine  Health Affairs  Health Services Research  Healthcare Executive  Healthcare Financial Management  JAMA | JAMA Internal Medicine  Journal of General Internal Medicine  Journal of Heatlhcare Management  Journal of Managed Care Pharmacy  Journal of the American Board of Family Medicine  Journal of the American Geriatrics Society  Journal of the American Medical Directors Association  New England Journal of Medicine  Obstetrics and Gynecology  Population Health Management  The American Journal of Managed Care  The Journal of Medical Practice Management  Value in Health |
| --- | --- |

**Use of Terms by Different Stakeholders**

In Figure A-3, *perspective* refers to the perspective of the authors, typically derived from the affiliation of the principal author as identified in the article.

**Figure A-3: Mix of Author Perspectives in Articles Selected for Definitions/Uses of *Population Health* and *Population Medicine***
