# Supplementary material for: Seeking Consensus on the Terminology of Value-Based Transformation Through use of a Delphi Process
Source: Popul Health Manag. 2020 Jun 3;23(3):243–55. doi: 10.1089/pop.2019.0093 (PMC7301322; doi:10.1089/pop.2019.0093)
Supplement: Supplemental data [file Suppl_TableS1-S3.pdf]

Supplementary Data

Supplementary Table S1. Delphi Survey Components and Ratings for *Value-Based Care*

How essential are components to include in the definition of this term? (1-not at all to 9-extremely)	*1-3 n (%)*	*4-6 n (%)*	*7-9 n (%)*	*Total n*	*Consensus*
**ROUND 1: COMPONENTS FOR *VALUE-BASED CARE***					
1. A definition of value should be included	0 (0.0)	2 (11,8)	15 (88.2)	17	Yes
2. Value is defined as health outcomes achieved per dollar spent	3 (16.7)	8 (44.4)	7 (38.9)	18	No
3. Reference to the Triple Aim of achieving quality outcomes, lowering costs, and improving the patient experience	7 (38.9)	4 (22.2)	7 (38.9)	18	No
4. Managing the health of populations	3 (316.7)	9 (50.0)	6 (33.3)	18	No
5. Improving health for populations	3 (17,.6)	4 (23.58)	10 (58.8)	17	Half support
6. Improving quality of care	2 (11.8)	3 (17.6)	11 (70.6)	17	Yes
7. Controlling costs	3 (16.7)	7 (38.9)	8 (44.4)	18	No
8. Improving health outcomes	0 (0.0)	0 (0.0)	18 (100)	18	Yes
9. Improving health for individuals	0 (0.0)	6 (33.3)	12 (66.7)	18	Approaching
10. Improving health for families	2 (11.1)	9 (50.0)	7 (38.9)	18	No
11. Improving the health of communities	3 (16.7	5 (27.8)	10 (55.6)	18	Half Support
12. Improving the patient experience	2 (11.2)	4 (22.2)	12 (66.7)	18	Approaching
13. Linking payment to cost objectives	10 (58.8)	4 (23.5)	3 (17.6)	17	No
14. Linking payment to quality objectives	10 (55.6)	4 (22.2)	4 (22.2)	18	No
15. Linking payment to health objectives	9 (50.0)	3 (16.7)	6 (33.3)	18	No
16. Providers or health systems assuming level of risk	10 (55.6)	6 (33.3)	2 (11,1)	18	No
17. Keeping people healthy	0 (0.0)	9 (50.0)	9 (50.0)	18	Half Support
**ROUND 2: COMPONENTS FOR *VALUE-BASED CARE***					
1. Improve the health of patients	0 (0.0)	2 (11.1)	16 (88.9)	18	Yes
2. Improve the health of populations	1 (5.6)	5 (27.8)	12 (66.7)	18	Approaching
3. Improve the health of communities	2 (511.1)	14 (77.8)	1 (11.1)	18	No
4. Improve the health of individuals and populations	1 (5.6)	4 (22.2)	13 (72.2)	18	Yes
5. Improve quality of care	1 (5.6)	5 (27.8)	12 (66.7)	18	Approaching
6. Include a definition of value	1 (5.6)	3 (16.7)	14 (77.8)	18	Yes
7. Numerator of value equation is health outcomes	0 (0.0)	8 (44.4)	10 (55.6)	18	Half Support
8. Numerator of value equation is health benefits	4 (22.2)	8 (44.4)	6 (33.3)	18	No
9. Further define benefits as quality of care, health outcomes, and the patient experience	3 (16.7)	7 (38.9)	8 (44.4)	18	No
10. Further define benefits as measurable processes of care and health outcomes	9 (50.0)	6 (33.2)	3 (16.7)	18	No
11. Improving objectively measurable aspects of patient experience	5 (27.8)	7 (38.9)	6 (33.3)	18	No
12. ''Patient-important outcomes'' as a term to encompass health outcomes and patient experience	6 (33.3)	6 (33.3)	6 (33.3)	18	No
13. Value includes affordable care	3 (16.7)	9 (50.0)	6 (33.3)	18	No
14. Value includes access to care	4 (22.2)	9 (50.0)	5 (27.8)	18	No
15. Value includes patient experience	3 (16.7)	8 (44.4)	7 (38.9)	18	No
16. Value includes preference aligned patient decisions	3 (16.7)	8 (44.4)	7 (38.9)	18	No
17. Value defined as what matters most to patients	2 (11.1)	11 (61.1)	5 (27.8)	18	No
18. Value is measured over a time horizon that exceeds individual episodes of care	0 (0.0)	9 (50.0)	9 (50.0)	18	Half Support
19. Value is focused on providing high-value care within the constraints of available resources	3 (16.7)	7 (38.9)	8 (44.4)	18	No
20. Goal of value-based care is to optimize our national health care system	10 (55.6)	4 (22.2)	4 (22.2)	18	No
21. Denominator of value equation: dollar spent	4 (22.2)	10 (55.6)	4 (22.2)	18	No
22. Denominator of value equation: cost	2 (11.1)	4 (22.2)	12 (66.7)	18	Approaching
23. Specify costs can be calculated from a variety of perspectives	5 (27.8)	7 (38.9)	6 (33.3)	18	No
24. Define care to include maintaining wellness, prevention, and treatment	5 (27.8)	5 (27.8)	8 (44.4)	18	No

(*continued*)

Supplementary Table S1. (Continued)

How essential are components to include in the definition of this term? (1-not at all to 9-extremely)	*1-3 n (%)*	*4-6 n (%)*	*7-9 n (%)*	*Total n*	*Consensus*
25. Define care to include addressing social determinants of health	2 (11.1)	10 (55.6)	6 (33.3)	18	No
26. Denominator of value equation: dollar spent	4 (22.2)	10 (55.6)	4 (22.2)	18	No
27. Define care as holistic	6 (33.3)	8 (44.4)	4 (22.2)	18	No
28. Define care as patient-centric	2 (11.1)	8 (44.4)	8 (44.4)	18	No
29. ''Care processes'' as a term to encompass quality of care and patient experience	7 (38.9)	7 (38.9)	4 (22.2)	18	No
30. Reference to triple aim of improving the patient experience of care, improving the health of populations, and reducing the per capita cost of health care.	4 (22.2)	8 (44.4)	6 (33.3)	18	No
**ROUND 3: COMPONENTS FOR *VALUE-BASED CARE***					
1. Value equation described as ''measurable health outcomes per cost of care''.	3 (16.7)	5 (27.8)	10 (55.6)	18	Half Support
2. Value equation described as ''measurable health outcomes relative to cost of care''.	2 (11.2)	4 (22.2)	12 (72.2)	18	Approaching
3. Value equation described as ''patient centered health outcomes achieved per cost of care''.	2 (11.2)	9 (50.0)	7 (38.9)	18	No
4. Value-based care is provided over an extended time horizon.	5 (27.8)	9 (50.0)	4 (22.2)	18	No
5. Value-based care is provided within the constraints of available resources.	5 (27.8)	11 (61.1)	2 (11.2)	18	No

Supplementary Table S2. Delphi Survey Components and Ratings for *Value-Based Payment*

How essential are components to include in the definition of this term? (1-not at all to 9-extremely)	*1-3 n (%)*	*4-6 n (%)*	*7-9 n (%)*	*Total n*	*Consensus*
**ROUND 1: COMPONENTS FOR *VALUE-BASED PAYMENT***					
1. A definition of value should be included	1 (5.9)	4 (23.5)	12 (70.6)	17	Yes
2. Contrast with fee-for-service payment	3 (16.7)	8 (44.4)	7 (38.9)	18	No
3. Contrast with volume-based payment	8 (44.4)	6 (33.3)	4 (22.2)	18	No
4. Rewarding healthcare providers	6 (33.3)	9 (50.0)	3 (16.7)	18	No
5. Improving quality of care	1 (5.6)	5 (27.8)	12 (66.7)	18	Approaching
6. Lowering costs per capita	6 (33.3)	5 (29.4)	7 (41.2)	18	No
7. Increasing efficiency of care	5 (29.4)	5 (29.4)	7 (44.4)	17	No
8. Improving the patient experience	1 (5.9)	7 (41.2)	9 (52.9)	17	Half support
9. Improving health outcomes	9 (50.0)	6 (33.3)	3 (16.7)	18	No
10. Engaging consumers to be knowledgeable consumers of healthcare	5 (27.8)	11 (61.1)	2 (11.1)	18	No
11. Reaching quality targets	7 (38.9)	9 (50.0)	2 (11.1)	18	No
12. Reaching cost targets	0 (0.0)	2 (11.1)	15 (88.2)	17	Yes
13. Utilization measures	9 (52.9)	7 (41.2)	1 (5.9)	17	No
14. Performance measures	9 (52.9)	8 (44.4)	6 (33.3)	18	No
15. Focus on population health	1 (5.9)	4 (23.5)	12 (70.6)	17	Yes
16. Financial incentives for healthcare providers	5 (27.8)	6 (33.3)	7 (38.9)	18	No
17. Accountability	1 (5.6)	5 (27.8)	12 (66.7)	18	Approaching
18. Incorporating risk sharing	4 (22.4)	6 (33.3)	8 (44.4)	18	No
**ROUND 2: COMPONENTS FOR *VALUE-BASED PAYMENT***					
1. A definition of value should be provided	0 (0.0)	0 (0-0)	18 (100)	18	Yes
2. Supporting value-based care	2 (11.1)	4 (22.2)	12 (66.7)	18	Approaching
3. Contrast with fee-for-service	1 (5.6)	8 (44.4)	9 (50.0)	18	Half support
4. Contrast with payment based on volume of services	1 (5.6)	8 (44.4)	9 (50.0)	18	Half support
5. ''Efficient'' to express benefits/cost or outcomes/cost	3 (16.7)	9 (50.0)	6 (33.3)	18	No
6. Spans care settings	4 (22.2)	7 (38.9)	7 (38.9)	18	no
7. Lowering costs per capita (as opposed to per patient)	5 (27.8)	10 (55/6)	3 (16.7)	18	No
8. ''Performance measures'' instead of ''goals''	5 (27.8)	8 (44/4)	5 (27.8)	18	No
9. Population level outcomes	1 (5.6)	6 (33.3)	11 (61.1)	18	Approaching
10. Individual level outcomes	1 (5.6)	7 (38.9)	10 (55.6)	18	Half-support
11. Incentives	4 (22.2)	7 (38.9)	7 (38.9)	18	No
12. Risk sharing	5 (27.8)	8 (44.4)	5 (27.8)	18	No

(*continued*)

Supplementary Table S2. (Continued)

How essential are components to include in the definition of this term? (1-not at all to 9-extremely)	*1-3 n (%)*	*4-6 n (%)*	*7-9 n (%)*	*Total n*	*Consensus*
13. Provider organizations assuming risk for cost of medical services	6 (33.3)	8 (44.4)	4 (22.2)	18	No
14. Accountability of providers for goals/outcomes/metrics	1 (5.6)	3 (16.7)	14 (77.8)	18	Yes
15. Outcomes based on longer time frame than a single episode of care	3 (16.7)	3 (16.7)	12 (66.7)	18	Approaching
16. Setting realistic goals	6 (33.3)	7 (38.9)	5 (27.8)	18	No
17. Reimbursement for healthcare	5 (27.8)	7 (38.9)	6 (33.3)	18	No
18. Reimbursement of healthcare delivery	4 (22.2)	9 (50.0)	5 (27.8)	18	No
19. Reimbursement for healthcare providers	6 (33.3)	6 (33.3)	6 (33.3)	18	No
20. Reimbursement for provider organizations	7 (38.9)	8 (44.4)	3 (16.7)	18	No
21. Provider experience	8 (44.4)	7 (38.9)	3 (16.7)	18	No
22. Provider teams	6 (33.3)	6 (33.3)	6 (33.3)	18	No
23. Process measures as surrogate for outcome measures	8 (44.4)	8 (44.4)	1 (11.1)	18	No
24. Increasing capacity to care for more patients	12 (66.7)	5 (27.8)	1 (5.6)	18	No
**ROUND 3: COMPONENTS FOR *VALUE-BASED PAYMENT***					
1. Providers held accountable for achieving financial goals and health outcomes.	1 (5.6)	5 (27.8)	12 (66.7)	18	Approaching
2. A shift from reimbursement for health care based on volume of services to outcomes-based reimbursement.	1 (5.6)	4 (22.2)	12 (72.2)	18	Yes
3. In value-based payment, outcomes are measured over a specified period of time.	2 (11.2)	7 (38.9)	9 (50.0)	18	Half-support
4. Value-based payment encourages optimal care delivery, including coordination across health care disciplines and between the health care system and community resources, to improve health outcomes for both individuals and populations.	2 (11.1)	4 (22,2)	12 (66.7)	18	Approaching

Supplementary Table S3. Delphi Survey Components and Ratings for *Population Health*

How essential are components to include in the definition of this term? (1-not at all to 9-extremely)	*1-3 n (%)*	*4-6 n (%)*	*7-9 n (%)*	*Total n*	*Consensus*
**ROUND 1: COMPONENTS FOR *POPULATION HEALTH***					
1. Social determinants of health	3 (16.7)	6 (33.3)	9 (50.0)	18	Half Support
2. Access to health care	2 (11.8)	9 (52.9)	6 (35.3)	17	No
3. Social environment	5 (27.8)	6 (33.3)	7 (38.9)	18	No
4. Physical environment	5 (27.8)	7 (38.9)	6 (33.3)	18	No
5. Health behavior	3 (18.8)	7 (43.8)	6 (37.5)	16	No
6. The distribution of health outcomes	1 (6.2)	5 (31.2)	10 (58.8)	16	Approaching
7. The health of a group of individuals	0 (0.0)	4 (25.0)	12 (75.0)	16	Yes
8. Recognizing the different population denominators (i.e., by geography, health system, health provider)	2 (11.8)	5 (29.4)	10 (58.8)	17	Half support
9. Improving the health of a population	2 (11.8)	3 (17.6)	12 (70.6)	17	Yes
10. Reducing disparities in health	2 (11.8)	4 (23.5)	11 (64.7)	17	Approaching
11. Increasing equity in health	1 (6.2)	5 (31.2)	10 (62.5)	16	Approaching
12. Improving quality of life	4 (23.5)	5 (29.4)	8 (47.1)	17	No
13. Improving functional status	4 (23.5)	4 (23.5)	9 (52.9)	17	Half support
14. Increasing the value of health care	3 (17.6)	4 (23.5)	10 (58.8)	17	Half support
15. Considering multilevel factors impacting health (health care policy, heath care systems, clinical practice, patient and community)	4 (23.5)	5 (29.4)	8 (47.1)	17	No
**ROUND 2: COMPONENTS FOR *POPULATION HEALTH***					
1. Social determinants of health	2 (11.1)	4 (22.2)	12 (66.7)	18	Approaching
2. A range of determinants such as social determinants (education, housing, environmental safety, food), genetic make-up, health behaviors, and access to care.	2 (11.1)	3 (16.7)	13 (72.2)	18	Yes
3. Access to health care as a determinant of population health.	3 (16.7)	7 (38.9)	8 (44.4)	18	No
4. Distribution of health outcomes	1 (5.6)	5 (27.8)	11 (66.6)	18	Approaching
5. The health of a group of individuals	2 (11.1)	4 (22.2)	12 (66.7)	18	Approaching
6. A population denominator can be defined in many ways.	4 (22.2)	5 (27.8)	9 (50.0)	18	Half Support

(*continued*)

Supplementary Table S3. (Continued)

How essential are components to include in the definition of this term? (1-not at all to 9-extremely)	*1-3 n (%)*	*4-6 n (%)*	*7-9 n (%)*	*Total n*	*Consensus*
7. Linking the importance of population health to the value-based care model.	5 (27.8)	7 (38.9)	6 (33.3)	18	No
8. List domains of health such as social, mental, and physical	1 (5.6)	10 (55.6)	7 (38.9)	18	No
9. Define health in terms of overall well-being	2 (11.1)	7 (38.9)	9 (50.0)	18	Half support
10. Include specific metrics of health such as functional status, quality of life, morbidity, and mortality	3 (16.7)	6 (33.3)	9 (50.0)	18	Half-support
11. Include social community as one type of population.	9 (50.0)	6 (33.3)	3 (16.7)	18	No
12. Reference policy as an influence on determinants of health (for example, both legislation ad payer coverage policies can affect access to health care).	7 (38.9)	6 (33.3)	5 (27.8)	18	No
**ROUND 3: COMPONENTS FOR *POPULATION HEALTH***					
1. Socioeconomic, environmental, biologic, and behavioral determinants.	3 (16.7)	8 (44.4)	7 (38.9)	18	No
2. A range of determinants, which might include socioeconomic, environmental, biologic, and/or behavioral factors.	3 (16.7)	6 (33.3)	9 (50.0)	18	Half Support
3. A group is defined by common characteristics such as geography, demographic factors, health conditions, or health care setting.	2 (11.1)	6 (33.3)	10 (55.6)	18	Half Support
4. Health encompasses social, mental, and physical well-being	3 (16.7)	7 (38.9)	8 (44.4)	18	No
